# The G-Protein–Coupled Estrogen Receptor Agonist G-1 Inhibits Proliferation and Causes Apoptosis in Leukemia Cell Lines of T Lineage

**DOI:** 10.3389/fcell.2022.811479

**Published:** 2022-02-14

**Authors:** Liliana Torres-López, Miguel Olivas-Aguirre, Kathya Villatoro-Gómez, Oxana Dobrovinskaya

**Affiliations:** Laboratory of Immunobiology and Ionic Transport Regulation, Centro Universitario de Investigaciones Biomédicas, Universidad de Colima, Colima, Mexico

**Keywords:** acute lymphoblastic leukemia, GPER agonist G-1, proliferation, apoptosis, cell cycle, microtubules

## Abstract

The G-protein–coupled estrogen receptor (GPER) mediates non-genomic action of estrogen. Due to its differential expression in some tumors as compared to the original healthy tissues, the GPER has been proposed as a therapeutic target. Accordingly, the non-steroidal GPER agonist G-1, which has often demonstrated marked cytotoxicity in experimental models, has been suggested as a novel anticancer agent for several sensitive tumors. We recently revealed that cell lines derived from acute T-cell (query) lymphoblastic leukemia (T-ALL) express the GPER. Here, we address the question whether G-1 is cytotoxic to T-ALL. We have shown that G-1 causes an early rise of intracellular Ca^2+^, arrests the cell cycle in G2/M, reduces viability, and provokes apoptosis in T-ALL cell lines. Importantly, G-1 caused destabilization and depolymerization of microtubules. We assume that it is a disturbance of the cytoskeleton that causes G-1 cytotoxic and cytostatic effects in our model. The observed cytotoxic effects, apparently, were not triggered by the interaction of G-1 with the GPER as pre-incubation with the highly selective GPER antagonist G-36 was ineffective in preventing the cytotoxicity of G-1. However, G-36 prevented the intracellular Ca^2+^ rise provoked by G-1. Finally, G-1 showed only a moderate negative effect on the activation of non-leukemic CD4^+^ lymphocytes. We suggest G-1 as a potential antileukemic drug.

## Introduction

Acute lymphoblastic leukemia of T lineage (T-ALL) represents an aggressive hematologic neoplasia with a significant morbidity rate in children and adults. Although ALL of immature B lymphocytes is of the highest incidence among ALL pediatric patients, T-ALL shows a higher rate of refractory and relapse in all age groups (Raetz and Teachey, 2016; Vadillo et al., 2018), being an important clinical problem and a challenge in search for new drug targets.

Estrogens are pleiotropic steroid hormones that contribute to the maturation and differentiation of the immune cells (Yakimchuk et al., 2013). Their immunomodulating effects as suppressors or activators of immune responses seem to depend largely on the cell type and cellular context (Khan et al., 2012; Kovats, 2015). The biological effects of estrogens can occur through nuclear estrogen receptors (ERs) and the G-protein–coupled ER (GPER, initially denominated as GPR30), which mediate rapid non-genomic cell signaling events (Filardo et al., 2000; Revankar et al., 2005). The GPER expression was revealed in most if not all normal tissues, and it has also been shown that the GPER plays an important role in the progression of various types of cancer, especially those dependent on hormones (Prossnitz and Barton., 2011; Jala et al., 2012; Girgert et al., 2019; Hsu et al., 2019; Jung, 2019; Xu et al., 2019). Some authors reported an increased GPER expression in neoplastic cells as compared to the original healthy tissue (Jala et al., 2012). There are several works linking the GPER expression or specific subcellular GPER localization with the outcome in cancer patients (Filardo et al., 2006; Sjöström et al., 2014; Tutzauer et al., 2020). GPER has been proposed as a novel therapeutic target in different cancer types (Ariazi et al., 2010; Wang et al., 2012; Lv and Wang, 2014; Weißenborn et al., 2014; Chimento et al., 2015; Kurt et al., 2015; Mori et al., 2015; Rudelius et al., 2015).

As estrogens were demonstrated to bind and modulate both the nuclear ER and GPER, specific GPER ligands were necessary for research purpose to dissect physiological effects attributed to each specific receptor type. Accordingly, the highly selective non-steroid GPER agonist G-1 (Bologa et al., 2006) and antagonists G-15 (Dennis et al., 2009) and G-36 (Dennis et al., 2011) have been developed. Numerous studies reported antiproliferative and cytotoxic effects of G-1 in different experimental models (Supplementary Table S1), and therefore, it was suggested as a possible candidate for anticancer therapies (Wang et al., 2012; Lv and Wang, 2014; Mori et al., 2015; Chimento et al., 2015; Kurt et al., 2015; Zhou et al., 2021). Eventually, it was noted that in different experimental systems G-1 displayed diverse and even opposite effects, sometimes suppressing cell proliferation and causing cell death or, on the contrary, inducing cell growth and proliferation (Supplementary Table S1). There are numerous studies that reported a specific GPER-dependent cytotoxic effect of G-1 in different cancer models (Albanito et al., 2007; Dennis et al., 2011; Imesch et al., 2013; Luo et al., 2014; Wei et al., 2014; Weißenborn et al., 2014; Cirillo et al., 2017; Ribeiro et al., 2017; Lee et al., 2019; Liu et al., 2019; Han et al., 2021; Zhou et al., 2021). But several studies have evidenced that G-1 is able to cross the plasma membrane and suppress cell viability through mechanisms independent from the GPER (Holm et al., 2012; Wang et al., 2012; Gui et al., 2015; Mori et al., 2015; Lv et al., 2017). There is also evidence that cytoskeleton microtubules may represent the main intracellular G-1 target and that the microtubule network is disrupted by G-1 treatment (Holm et al., 2012; Wang et al., 2013; Lv et al., 2017).

Hematological malignances are not considered to be estrogen-dependent. However, it is very likely that they are influenced by estrogens because gender differences in their incidence and prognosis were reported (Yakimchuk et al., 2013). There is accumulated evidence for the ER expression in lymphoid progenitors, healthy and malignant lymphocytes, and for the role which they play in immune system physiology and in the pathogenesis of different lymphoproliferative disorders (Catusse et al., 2010; Yakimchuk et al., 2013; Rudelius et al., 2015; Ladikou and Kassi., 2017; Hasni and Yakumchuk., 2019). Modulation of hematopoietic progenitors by estrogens was suggested as a basis for novel antileukemic strategies for acute myeloid leukemia (Sánchez-Aguilera and Méndez-Ferrer, 2016).

We have recently demonstrated that healthy activated T lymphocytes express both the nuclear ER and GPER. In contrast, lymphoblasts of cell lines derived from male and female T-ALL patients preferentially expressed the GPER (Torres-López et al., 2019). The present work was designed to evaluate the biological effects of the selective GPER agonist G-1 in the T-ALL Jurkat cell line.

## Materials and Methods

### Cell Lines and Culture Conditions

Human T-ALL cell lines Jurkat (clone E6-1, ATCC^®^TIB™, male, 14 years); MOLT-3 (ATCC^®^CRL-1552™, male, 19 years), and CCRF-CEM (ATCC^®^CCL-119™, female, 4 years) were purchased from ATCC. Cells were cultured in suspension in an Advanced RPMI 1640 medium supplemented with 5% (v/v) heat-inactivated fetal bovine serum (FBS), 2 mM GlutaMAX, 100 U/ml penicillin, 100 μg/ml streptomycin, and 10 mM HEPES (all from Gibco, Thermo Fisher Scientific, Waltham, MA, United States). In some experiments, Jurkat cells were maintained in a phenol red–free RPMI 1640 medium supplemented with 10% (v/v) heat-inactivated dialyzed fetal calf serum (FCS, Gibco). Adherent breast cancer cell lines MDA-MB-231 (ATCC^®^HTB-26™) and MCF-7 (ATCC^®^ HTB-22™) were cultured in the DMEM supplemented with 10% (v/v) of heat-inactivated FBS, 100 U/ml of penicillin, 100 μg/ml streptomycin, and 1% of GlutaMAX. All cell lines were maintained in a logarithmic growth phase by medium refreshment, in a humidified incubator in atmosphere with 5% CO_2_ at 37°C.

### Chemicals

G-1, 1-[(3aS,4R,9bR)-4-(6-bromo-1,3-benzodioxol-5-yl)-3a,4,5,9b-tetrahydro-3Hcyclopenta[c]quinolin-8-yl] ethanone (10008933) and G-36, (3aS,4R,9bR)-4-(6bromo-1,3-benzodioxol-5-yl)-8-propan-2-yl-3a,4,5,9b-tetrahydro-3H-cyclopenta[c] quinoline (14397), both from Cayman Chemical, were dissolved in DMSO. Typically, stock solutions were prepared at a concentration of 20 mM and stored at −20°C. Working solutions were prepared from stock solutions in a growth medium immediately before the experiments. The effect of the vehicle (DMSO at 0.05% v/v), corresponding to the highest used concentrations of G-1 and G-36 (10 μM), was tested in all protocols and showed no effect. 2-aminoethyl diphenylborinate (2-APB, D9754), carbonyl cyanide 4-(trifluoromethoxy) phenylhydrazone (FCCP, C2920), and phorbol 12-myristate 13-acetate (PMA, P8139) were purchased from Sigma-Aldrich. 2-APB and FCCP were dissolved in ethanol, while PMA was dissolved in DMSO.

### Purification and Activation of Primary CD4^+^ Lymphocytes

Peripheral blood mononuclear cells (PBMCs) were isolated by the Ficoll-Paque method (Ficoll-Paque PLUS, 17-1440-02, GE Healthcare) from heparinized blood of apparently healthy donors. The protocol was approved by the Bioethics and Biosecurity Committee of the Biomedical Research Centre in accordance with federal (Artículo 100, Ley General de Salud), state, and local laws. PBMCs were subjected to negative selection, to avoid untimely activation, using a human CD4^+^ T-cell isolation kit (130-096-533, Miltenyi Biotec) following manufacturers’ specifications. For synchronous polyclonal activation, 1 × 10^5^ resting CD4^+^ lymphocytes were seeded in 24-well plates pretreated with anti-CD3 monoclonal antibodies (BD 555336, 5 μg/ml in phosphate-buffered saline solution (PBS), 2 h at 37°C) and incubated with anti-CD28 monoclonal antibodies (BD 555725, 2 μg/ml in RPMI 1640 Medium) for 4 days. Co-stimulation with CD3/CD28 provides a potent antigen-independent activating stimulus by cross-linking T-cell receptors (TCR) of resting T lymphocytes.

### Fluorescent Immunocytochemistry

To evaluate subcellular GPER localization, trypsinized adherent MCF-7 and MDA-MB-231 cells and suspension cells were washed with PBS and fixed on silanized glass slides with ice-cold 100% methanol for 5 min at room temperature, washed three times again, and incubated for 30 min in the permeabilization/blocking buffer (1% of bovine serum albumin: BSA, 22.52 mg/ml glycine and 0.1% Tween 20 dissolved in PBS). After this, cells were incubated with primary anti-human GPER rabbit antibodies (Novus Biologicals NBP1-31239, dilution 1:200) and then with Texas Red–conjugated goat anti-rabbit IgG (Abcam ab7088, dilution 1:100), as secondary antibodies. For Na^+^/K^+^- ATPase detection, primary anti-human Na^+^/K^+^—ATPase α1 mouse antibodies (Santa Cruz Biotechnology sc-21712, dilution 1:100) and secondary Chromeo™ 488-conjugated goat anti-mouse IgG antibodies (Abcam ab60313, dilution 1:1500) were used. For visualization of microtubules, primary mouse anti-human α-tubulin monoclonal antibodies conjugated with Alexa Fluor 488 were used (Invitrogen 32-2588, dilution 1:100). All antibodies were diluted in a PBS-T (PBS-0.1% Tween 20) solution containing 1% BSA, and samples were incubated in a humidified chamber for 1-2 h at room temperature. A ProLong™ Gold Antifade mountant was used for mounting samples (Invitrogen P36934). Slides were observed by confocal microscopy. Two sets of microscopes were used: A Carl Zeiss LSM 700 system coupled to an inverted Observer Z1 microscope equipped with 488 and 555 nm lasers and with 40x objective or, alternatively, a custom-made confocal microscope (Solamere Technology Group, Salt Lake City, United States) based on a Yokogawa spin disk confocal scan head (CSUX1M1, Yokogawa Electronic Co., Tokyo, Japan), equipped with solid state Coherent Obis lasers (405, 488, 561, and 640 nm) and 100x objective was used. Images were analyzed by ZEN lite software 3.0 (ZEISS) and ImageJ software (National Institutes of Health). To assess microtubule integrity, the corrected total cell fluorescence (CTCF) was calculated, as described previously (Shakya et al., 2018) by the following formula:

CTCF= Integrated Density −(Area of selected cell∗ mean fluorescence of background readings) .

### Cell Viability Assays

Cell viability was evaluated using a resazurin-based metabolic *in vitro* toxicology assay kit (Sigma TOX-8) and by live cell count (trypan blue dye exclusion test). Cells (1 × 10^5^) were seeded in 96-well plates in 180 μl of RPMI per well. When specified, samples were pretreated with G-36 (10 μM, 30 min). The vehicle (DMSO) or G-1 in different concentrations were added, and cells were cultivated for 4–72 h, as indicated. For metabolic assay, 20 μl of the resazurin buffer was added to each well to complete volume to 200 μl, and plates were incubated 4 h at 37°C. To evaluate resazurin (non-fluorescent) to resorufin (fluorescent, Ex/Em max = 560/590 nm) conversion, a GloMax^®^Discover Multimode Microplate reader (Cat. GM3000, Promega) was used. Samples were run in triplicates, in at least three independent experiments. RPMI medium fluorescence was subtracted for each condition, and data obtained from resorufin fluorescence were averaged, normalized to their controls, and expressed as % of cell viability.

### CFSE-Based Cell Proliferation Assay

Carboxy-fluorescein succinimidyl ester (CFSE)-based cell proliferation assay (ThermoFisher C34554) with some modifications was used (Quah et al., 2007; Torres-López et al., 2019). Cells (1 × 10^6^) were washed with PBS and resuspended in 1 ml of CFSE-contained PBS, in a final concentration of 0.5 μM or 1 μM for cell lines and CD4^+^ lymphocytes, respectively. To ensure uniform staining of cells in the population, the samples were incubated for 30 min at room temperature with agitation by inverting the tube every 5 min. After the completion of staining, the cells were seeded in 24-well plates in a fresh, complete growth medium (5 × 10^5^/well) with a drug (vehicle or different concentrations of G-1) and cultivated during different time periods. CD4^+^ lymphocytes were stained with CFSE and then activated, as described previously. G-1 was added after 24 h, and the further activation continued in its presence. At set intervals, cells were harvested, washed in PBS, and CFSE fluorescence intensity was measured by flow cytometry (FACSCanto II, BD Biosciences). A total of 10,000 events in the live cell gate were collected for every sample. Data analysis was performed by FlowJo 10.2 Software. The median fluorescence intensity (MFI) of treated populations was normalized to control for leukemic cell lines, while the proliferation index was calculated for CD4^+^ lymphocytes.

### Monitoring of Cell Cycle Progression

Cells were treated with G-1 for 24–48 h. After that, cells were counted, washed with cold PBS, and fixed with ice-cold 70% ethanol overnight. The next day, cells were washed twice with PBS and permeabilized with 0.1% Triton X-100 in PBS (15 min). Finally, 500 μl of the PI working solution was added to each 1 × 10^6^ cells and incubated at least 24 h at 4°C. The composition of the working solution was as follows: 0.15 μM PI (Invitrogen, PNN1011), 10 μg/ml RNase (Sigma, R4875), and 0.1% BSA (Research Organics, 1336A) in PBS. The DNA content was evaluated by flow cytometry (FACSCanto II, BD Biosciences). For this, the PI fluorescence intensity (Ex/Em max = 538/617 nm) was measured, where 50,000 events in the intact cells gate were collected in each sample. Basing on the DNA content, the peaks corresponding to G1 and G2 cell cycle phases were determined. To exclude debris, appropriate gating of cell population in FSC versus SSC was made, following by gating in the PI-area versus PI-weight plot to exclude doublets. Data analysis was performed with ModFit 5.0 Software in trial version. Sub-G1 events were excluded from the analysis of cell cycle progression.

### Apoptosis/Necrosis Assay

To evaluate the type of cell death after treatments, a dead cell apoptosis kit (Invitrogen V13245) was used, following manufacturer’s specifications. Briefly, for each sample, 1 × 10^6^ cells were washed and stained in 100 μl of 1X annexin binding buffer containing 3 μl Annexin V-Alexa Fluor 488 (Ex/Em max: 488/510 nm) and 0.2 μl PI (Ex/Em max= 535/617 nm) for 15 min protected from light. Cells were analyzed by flow cytometry (FACSCanto II, BD Biosciences). The compensation procedure (Alexa Fluor 488 vs PI) was performed previously for data acquisition. For excitation, a 488 nm laser was used. PI fluorescence was measured using a 556LP mirror and a 585/42 filter; Alexa Fluor 488 fluorescence was measured using a 502LP mirror and a 530/30 filter. Debris and doublets were gated out, and 10,000 events in the gate of single cells per sample were collected. Autofluorescence control was used to settle on the threshold for positive fluorescence. Populations were classified as viable (Annexin V-Alexa Fluor 488 and PI negative, or double negative), apoptotic (Annexin V-Alexa Fluor 488 positive, PI negative), necrotic (Annexin V-Alexa Fluor 488 negative, PI positive), and late apoptotic/necrotic (Annexin V-Alexa Fluor 488 and PI positive, or double positive). Data analysis was performed with FlowJo 10.2.

### Measurements of Intracellular Free Ca^2+^ Concentration

For intracellular free Ca^2+^ concentration ([Ca ^2+^]_i_) measurements, leukemic cells were loaded with the intensiometric Ca^2+^ indicator Fluo 4-AM (Thermo Fisher Scientific Cat. F14201). Briefly, the cells were counted, collected, washed twice with PBS, and resuspended in Hanks balanced salt solution (HBSS, containing NaCl 143 mM, KCl 6 mM, MgSO_4_ 5 mM, HEPES 20 mM, BSA 0.1%, glucose 5 mM, CaCl_2_ 1.5 mM, *pH* = 7.4, ≈300 mOsm) and 2 µM of Fluo 4-AM (Ex/Em max of the Ca^2+^-bound form = 494/506 nm). Cells in HBSS were incubated for 30 min at room temperature in dark. Then, cells were washed to remove excessive (not incorporated) dye and resuspended in HBSS. Measurements were performed using a F7000, HITACHI spectrofluorometer (Hitachi High Technologies). Samples were placed in quartz cuvettes (1 × 10^6^ cells /ml), and data acquisition was performed every 2.5 s by exciting the samples at 488 nm and collecting emitted fluorescence at 510 nm. For Ca^2+^- free conditions, Ca^2+^-free HBSS containing 1 mM of EGTA was used. Data were normalized to initial fluorescence.

### Evaluation of Mitochondrial Membrane Potential Changes

To evaluate changes of the mitochondrial membrane potential (ΔΨm), the fluorescent dye rhodamine-123 (Rhod-123, Sigma R8004) was used. Binding of Rhod-123 (Ex/Em max = 507/530) to the mitochondria is directly proportional to ΔΨm. After treatments, cells were counted (trypan blue exclusion test), harvested (2.5 × 10^5^ cells), washed with PBS, and stained in 100 μl PBS containing 2 μM Rhod-123 (30 min at room temperature). Uncoupler FCCP (2 μM) was used as a positive control of mitochondrial membrane depolarization. To exclude dead necrotic cells, a double staining of Rhod-123 with PI (Dead Cell Apoptosis Kit, Invitrogen V13245) was performed. Changes in ΔΨm in PI-negative cells were estimated by flow cytometry (FACSCanto II, BD Biosciences). For excitation, a 488 nm laser was used. Rhod-123 fluorescence was measured using a 502LP mirror and 530/30 filter. A total of 10,000 events were collected in the gate of live cells for each sample. Data analysis was performed by FlowJo 10.2 Software.

### Measurement of ROS Production

To measure reactive oxygen species (ROS) production, cell-permeable 2’,7’-Dichlorofluorescin diacetate (DCFDA, D6883, Merck) was used following manufacturer’s recommendations. The method is based on the fact that the dye DCFH-DA is deacetylated by cellular esterases to a non-fluorescent compound which is later oxidized by ROS into its fluorescent form DCF (Ex/Em max = 495/529 nm). PMA (5 μM) was used as a positive control. After 1–2 h of treatment, Jurkat cells (5 × 10^5^) were washed with PBS and incubated with 5 μM DCFH-DA for 30 min at 37°C. DCF fluorescence in individual cells was estimated by flow cytometry (FACSCanto II, BD Biosciences). For excitation, a 488 nm laser was used, and the fluorescent signal was recollected using a 502LP mirror and 530/30 filter. In total, 10,000 events were collected for each sample. Data analysis was performed with FlowJo 10.2 software, and MFI was normalized to control.

### Data Analysis and Statistics

All experiments were performed at least three times (n ≥ 3) in an independent manner. Data were analyzed by GraphPad Prism 8.3 software and were expressed as mean values ± standard error (SEM). We compared the mean values in individual treatments with their control or, for the indicated experiments, the mean values between different treatments. The statistical significance was obtained with one-way analysis of variance (ANOVA) for comparison within the same group or two-way analysis for comparison between groups, with a *post hoc* analysis of Tukey for comparison between mean values of different groups, Dunnett for comparison of means values in respect to the control, or Sidak to compare the mean values of two different independent columns. The statistical significance was defined as *p* < 0.05.

## Results

### G-1 Suppresses Proliferation and Induces Apoptosis in T-ALL Cell Lines

**FIGURE 1:**
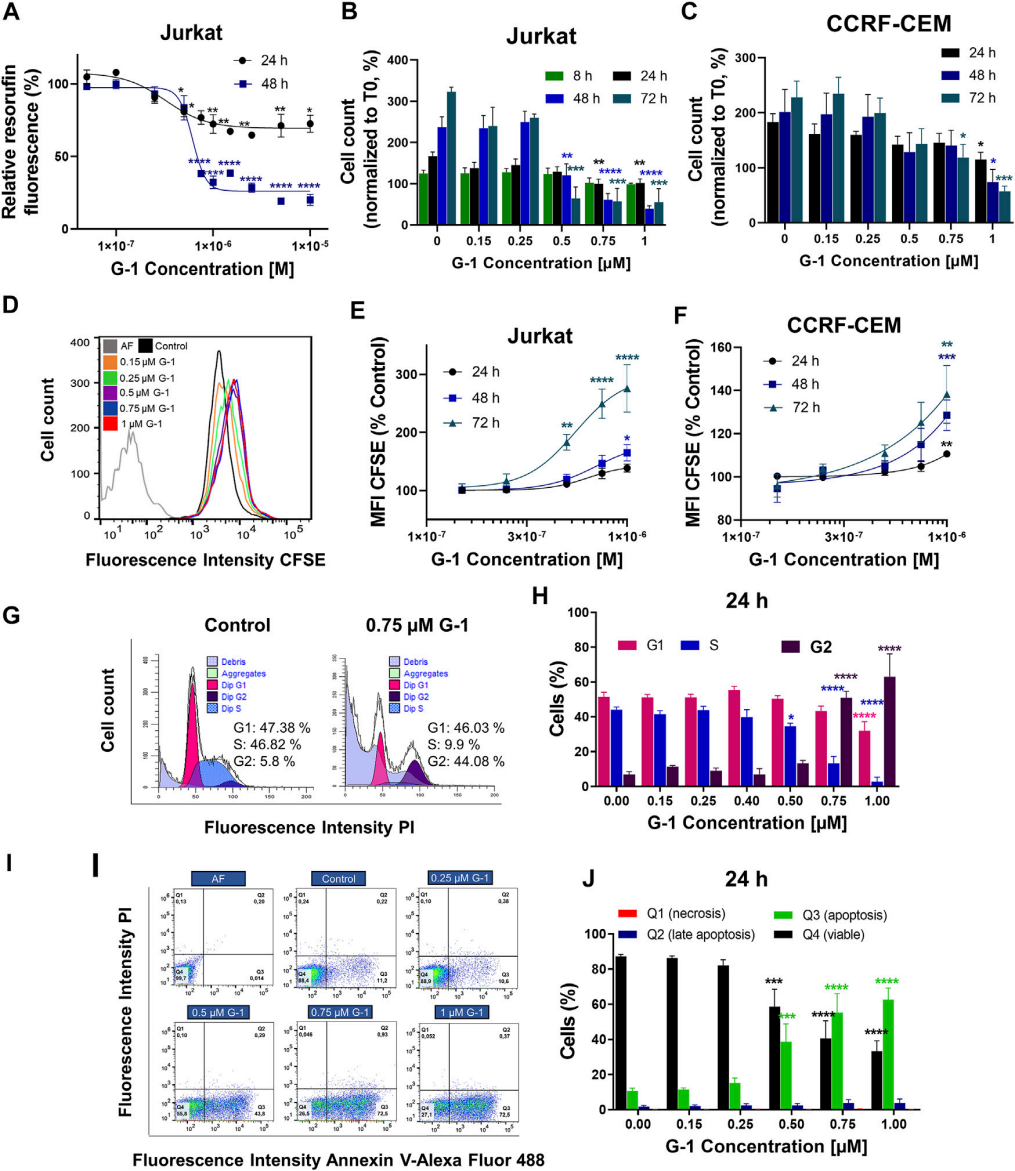
G-1 possesses antileukemic properties. Cells were incubated in the presence of growing concentrations of G-1 for the indicated periods of time. **(A)** Cell viability was evaluated by the resazurin-based metabolic assay, as a function of G-1 concentration. Data (resorufin fluorescence intensity, arbitrary units) of treated samples are normalized to the vehicle-treated control. **(B,C)** Cell viability was evaluated by live cell count (trypan blue exclusion test) at indicated time points. Data are normalized to the initial time point (0 h). **(A–C)** Data are mean ± SEM; N ≥ 3; *p < 0.05, **p < 0.01, ***p < 0.001, ****p < 0.0001; one-way ANOVA with Dunnett *post hoc* testing. **(D)** A representative histogram of changes in the CFSE fluorescence intensity of Jurkat cells growing in the presence of increasing concentrations of G-1 after 72 h of treatment; the shift of the mean fluorescence peak (MFI) to the left reflects cell proliferation. The autofluorescent population (AF, without CFSE staining) is shown. **(E,F)** MFI of CFSE of Jurkat **(E)** and CCRF-CEM **(F)** cells treated with G-1 normalized to control (untreated) cells as a function of G-1 concentration. Data are mean ± SEM; N ≥ 3; *p < 0.05, **p < 0.01, ***p < 0.001, ****p < 0.0001; one-way ANOVA with Dunnett *post hoc* testing. **(G)** Representative ModFit histograms of untreated cells and cells incubated with G-1 (0.75 μM, 24 h). The G0/G1 peak is pink, G2/M peak is purple, S phase between G0/G1 and G2/M is blue, and subG1 damaged population is light-purple. **(H)** Bar charts showing the percentage of the Jurkat cells in different subpopulations corresponding to G1, S, and G2 phases at 24 h of G-1 treatment. **(I)** Representative dot plots obtained by flow cytometry of Jurkat cells treated with G-1 during 24 h. Populations are as follows: Q1 necrotic (Annexin V/AlexaFluor488^−^ PI^+^), Q2 late apoptotic/necrotic (Annexin V/AlexaFluor488^+^PI^+^), Q3 apoptotic (Annexin V/AlexaFluor488^+^PI^−^), and Q4 viable (Annexin V/AlexaFluor488^−^PI^−^). **(J)** Bar charts showing the percentage of cells in Q1–Q4 population after 24 h of G-1 treatment. **(H,J)** Data are mean ± SEM; *N* = 3; *p < 0.05, ***p < 0.001, ****p < 0.0001. Comparison between control (without treatment) and G-1–treated samples was made by two-way ANOVA with Dunnett *post hoc* testing.

Comparative expression patterns of the ER in healthy and leukemic T lymphocytes were reported by our group recently, both on mRNA and protein levels (Torres-López et al., 2019). While healthy T cells express both the nuclear ER and GPER, leukemic ones preferentially express GPER localized, especially at the cell periphery, including the plasma membrane (Torres-López et al., 2019; Supplementary Figure S1). Taking in mind that G-1 can trigger different, sometimes opposite, cellular events depending on cell types (Supplementary Table S1), first we decided to prove whether G-1 is cytotoxic for T-ALL cell lines, Jurkat, and CCRF-CEM. Two widely recognized complementary tests were used, namely, resazurin-based metabolic assay (Figure 1A) and live/dead cell count (the trypan blue exclusion test, Figures 1B,C). For metabolic assay, Jurkat cells were cultured in the presence of G-1 in the range of 0.05–10 μM, as was reported for other cellular types (Supplementary Table S1), during 24 or 48 h. We observed that G-1 was toxic to Jurkat cells at concentrations ≥0.5 µM, and the highest effect was achieved at 1 µM (Figure 1A). Therefore, for further studies, the concentrations of up to 1 μM were chosen. G-1 caused a concentration-dependent reduction in both Jurkat and CCRF-CEM live cell counts, with the CCRF-CEM cell line being slightly less sensitive (Figures 1B,C). Considering the biological effects of 17-β-estradiol on Jurkat cells (Jenkins et al., 2001; Yedjou et al., 2015) and the weak estrogen-like activity of phenol red (Berthois et al., 1986), some additional experiments were undertaken in the phenol red–free RPMI medium supplemented with estrogen-free dialyzed FCS. Under these conditions, G-1 decreased the cell viability with similar EC_50_ for both conditions (Supplementary Figures S2A–D, Supplementary Table S2). Reduction in metabolism and the cell number may be caused by a decrease in the proliferation rate and/or induction of cell death. Thus, the contribution of both these processes to the observed cytotoxicity of G-1 was further addressed.

Classical proliferation assay based on cell staining with a nontoxic fluorescent cell tracker CFSE, adapted for continuously proliferating cell lines, was performed (Torres-López et al., 2019). In this assay, the cell tracker is equally distributed between two daughter cells during cell division, resulting in halving of the fluorescence intensity of individual cells, which can be evaluated by flow cytometry. In a dividing cell population, the histogram of CFSE fluorescence intensity is shifted to the left when compared to initial values (Figure 1D). If the drug has an antiproliferative effect, this shift is reduced. In our experiments, CFSE-stained Jurkat and CCRF-CEM cells were cultured in the presence of growing G-1 concentrations and evaluated by flow cytometry every 24 h for 3 days. In both cell lines, G-1 caused an antiproliferative effect, in some degree more pronounced in Jurkat cells (Figures 1E,F). We also analyzed the cell cycle progression in Jurkat cells, to reveal which phase of the cell cycle is being delayed. For this, the DNA content at the single-cell level was estimated by flow cytometry in populations of Jurkat cells, treated with different concentrations of G-1. Representative cell cycle histograms obtained in the control and in the G-1-treated (0.75 μM) populations at 24 h of culture are shown at the Figure 1G. We observed an increased event number in the Sub-G1 interval (DNA content less that in G1 phase), indicating cell death. Accordingly, cell cycle analysis was performed in the population of intact cells. Notably, in this population, the G1 peak and events in the S phase were decreased, whereas the G2 peak was increased. The percentage distribution of cell cycle phases in intact cells was determined at different G-1 concentrations (Figure 1H). One can appreciate that treatment with G-1, starting with 0.5 μM, caused a significant cell accumulation in the G2 phase. A similar effect was observed in Jurkat cells cultured in a phenol red–free RPMI medium (Supplementary Figures S2E,F). Next, Annexin V/PI assay was performed to determine the type of cell death caused by G-1. We have observed that G-1 effectively caused apoptosis in Jurkat cells (Figures 1I,J; Supplementary Figures S2G–I). Taken together, our data prove that G-1 causes cell cycle arrest in the G2 phase and subsequent apoptotic cell death.

### GPER Antagonist G-36 Did Not Prevent Cytotoxic Effects of G-1

**FIGURE 2:**
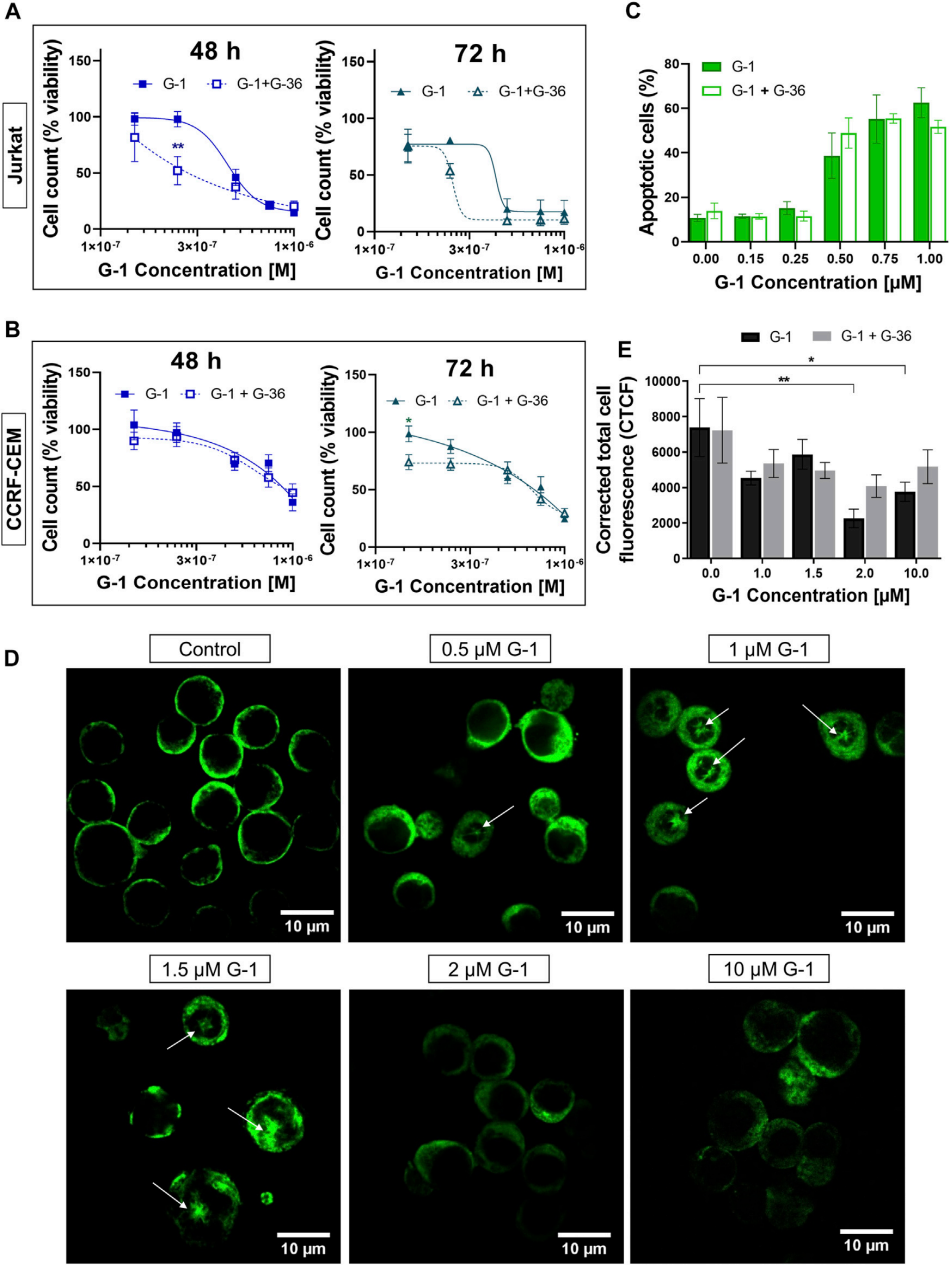
G-36 does not prevent antileukemic effects of G-1. **(A,B)** Jurkat **(A)** and CCRF-CEM **(B)** cells were pre-incubated with G-36 (10 μM, 30 min), seeded in a growth medium containing different concentrations of G-1 and cultivated during indicated periods of time. Cell viability was evaluated by cell count (trypan blue exclusion test) and graphed as function of G-1 concentration. Data are normalized to control and shown as mean ± SEM; N ≥ 3; *p < 0.05, **p < 0.01. Comparison between G-1 (solid line) and G1 + G-36 (dashed line) was performed by two-way ANOVA with Sidak *post hoc* testing. **(C)** Percentage of apoptotic cells (flow cytometry, Q3 population of Annexin V/AlexaFluor488^+^PI^−^ cells) at 24 h of treatment. Data are mean ± SEM; N ≥ 3; Comparison between G-1 and G-1 + G-36 groups was performed by two-way ANOVA with Sidak *post hoc* testing. No statistical difference was revealed. **(D)** Immunostaining of α-tubulin (green) in Jurkat cells treated with different concentrations of G-1 during 24 h. Representative images obtained by laser scanning confocal microscopy and processed by ImageJ software. Mouse antihuman α-tubulin monoclonal antibodies conjugated with Alexa Fluor 488 were used. White arrows indicate microtubule asters. Scale bar: 10 μm. **(E)** Bar charts showing the mean ± SEM of corrected total cell fluorescence. Mean fluorescence of ∼5–20 cells per field was evaluated; at least two fields per sample were analyzed in a minimum of three independent experiments. The means of each sample were compared to their control using a two-way ANOVA and a Dunnett *post hoc*, *p < 0.05, **p < 0.01. Similarly, each G-1 data was compared with the corresponding G-1 + G-36 one using two-way ANOVA and the Sidak *post hoc* test, but no statistically significant difference was obtained.

In order to reveal whether the cytotoxic effects of G-1 are triggered through GPER activation, the pharmacological blockage of this receptor by pretreatment with selective antagonists G-15 or G-36 is commonly used (see Supplementary Table S1 for references). G-36 has a higher selectivity toward the GPER than G-15 (Dennis et al., 2011). Additionally, no significant effect of G-36 on cell viability has been reported (Moreno-Ulloa, et al., 2018; Torres-López et al., 2019; Zhou et al., 2021), in contrast to G-15, which by itself can reduce cell growth (Bai et al., 2013; Imesch et al., 2013; Mori et al., 2015). In our previous study, pre-incubation with G-36 (10 μM, 30 min) efficiently prevented the autophagy induced by tamoxifen in Jurkat cells (Torres-López et al., 2019). Cell viability, proliferation, and apoptosis assays were performed in the same manner, as described above, but cells were pre-incubated with G-36 (10 μM, 30 min), then G-1 was added, and cells were cultured as usual in the presence of both drugs. First, G-36 itself did not affect cell proliferation and was not cytotoxic for Jurkat and CCRF-CEM cell lines (Supplementary Figures S3A–E). Contrary to expectations, G-36 neither prevented toxicity nor prevented antiproliferative effects of G-1 (Figures 2A–C; Supplementary Figures S3F–K). Interestingly, G-36-potentiated cytotoxicity of G-1, but only in the growth medium with phenol red supplemented with FBS (Figures 2A,B, Supplementary Figure S3J), and more evidently in Jurkat cells (see EC_50_ values in Supplementary Table S3). Our finding evidenced that the G-1 cytotoxicity against T-ALL cells is most likely triggered by mechanisms independent of the GPER, similar to some previous reports (Holm et al., 2012; Wang et al., 2012; Gui et al., 2015; Mori et al., 2015; Lv et al., 2017).

### G-1 Disrupts the Microtubule Structure in Jurkat Cells

Originally, G-1 was developed as a selective GPER agonist that did not bind to the nuclear ER (Bologa et al., 2006). But being fat-soluble and permeable through the plasma membrane, G-1 can bind to other intracellular molecular structures and cause the so named “off-target” effects. Some research groups reported the ability of G-1 to destroy the microtubules in different cell models, including ovarian adenocarcinoma (Wang et al., 2013), mantle B cell lymphoma (Rudelius et al., 2015), and breast cancer (Lv et al., 2017). Therefore, it was intriguing to evaluate, whether G-1 produces a change in the microtubule structure in leukemic cells. Figure 2D shows representative laser scanning confocal microscopy images of Jurkat cells, treated with increasing concentrations of G-1 (24 h), fixed and immunostained for α-tubulin. The normal morphology of untreated Jurkat lymphoblasts is characterized by their round shape, big nucleus with a high nucleus-to-cytoplasm ratio, and fine filaments of microtubules, arrayed in the cytoplasm. After treatment with G-1, the microtubule structure was changed dramatically. Remarkably, the pattern of changes was different in samples, treated with different concentrations of G-1. First of all, multiple microtubule asters were observed in cells treated with lower (0.5–1.5 μM) concentrations. Asters are formed during the prophase of mitosis, and their accumulation evidences the suppression of normal microtubule dynamics, which is necessary for progression through mitosis phases and cellular division. This phenomenon has been previously reported in ovarian cancer (Wang et al., 2013) and triple-negative breast cancer (Lv et al., 2017) cell lines, treated with G-1. Similar to our observations (Figures 1G,H), the cells were accumulated in the G2/M phase of the cell cycle due to the impossibility to form the mitotic spindle. G-1 was determined to attach to the colchicine binding site of tubulin (Lv et al., 2017) and can be considered as a colchicine analog. At a high concentration, colchicine analogs induce depolymerization of microtubules (Stanton et al., 2011). Indeed, at higher concentrations of G-1 (2–10 μM), the long microtubule fibers were completely disrupted and what was visualized as diffusely distributed staining was accompanied by a significant decrease in the fluorescence intensity. Then, we decided to measure total fluorescence of individual cells, as an indicator of microtubule integrity, as it was proposed earlier (Kasioulis et al., 2017; Shakya et al., 2018). At high concentrations of G-1, a significant decrease of CTCF was observed (Figure 2E). Cell appearance has also changed markedly; both the cells and their nuclei shrunk and the nucleus-to-cytoplasm ratio decreased. To reveal whether the effect was mediated by the GPER, some cultures were pre-treated with the GPER antagonist G-36, as described earlier. Pre-incubation with G-36 did not prevent the attenuation of the fluorescence intensity provoked by G-1 (Figure 2E). Accordingly, the observed effect of microtubule depolymerization was more likely caused by direct interaction of G-1 with tubulin and was not mediated by the interaction with the GPER.

### G-1 Induced Rapid Intracellular Calcium Rise in a GPER-Dependent Manner

**FIGURE 3:**
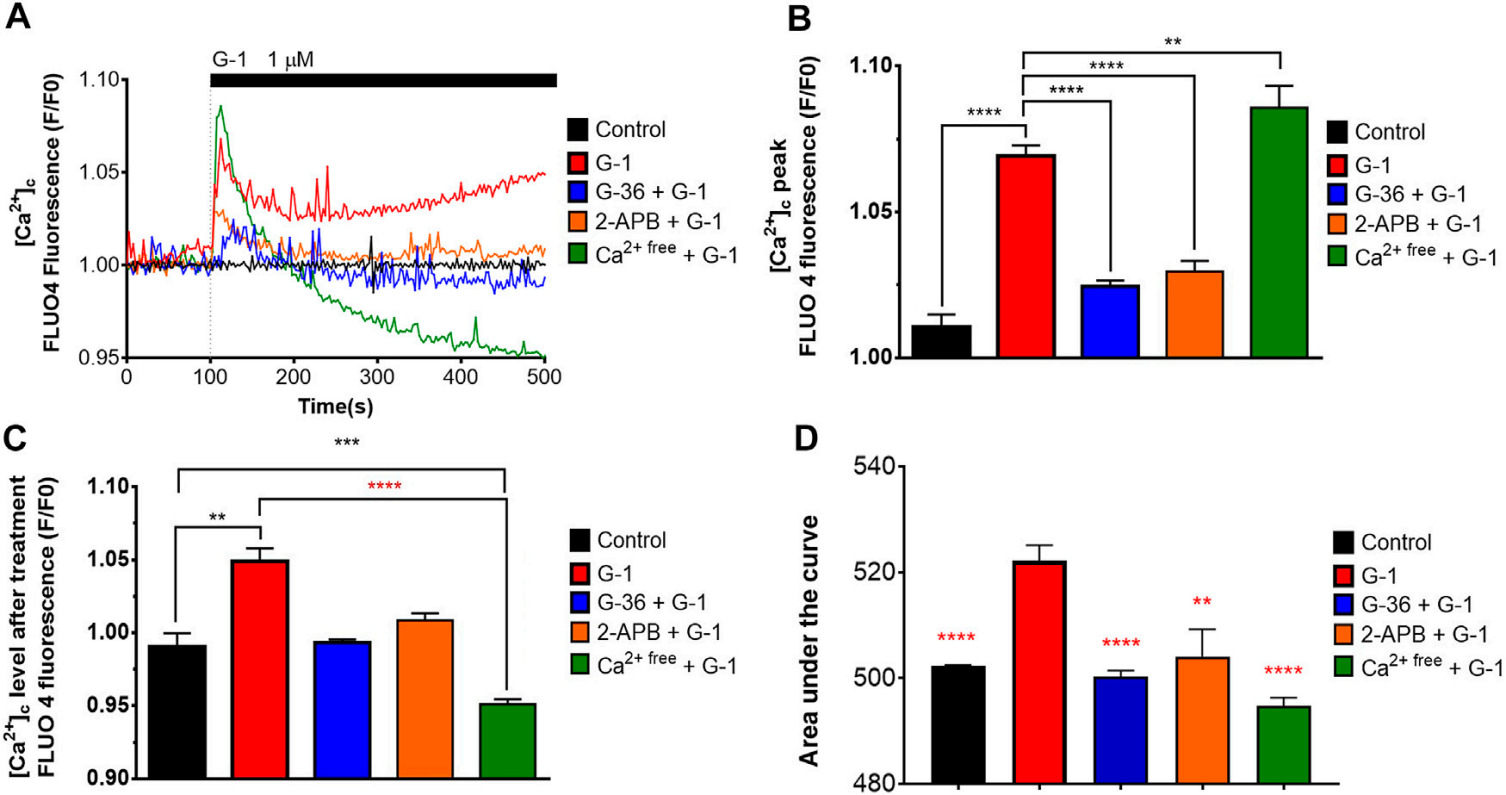
Cytosolic Ca^2+^ rise induced by G-1 in Jurkat cells is mediated by the GPER. **(A)** [Ca^2+^]_c_ monitoring in Jurkat cells loaded with Fluo 4. The time of G-1 injection is indicated. Traces represent the mean of at least six samples from three independent experiments. **(B–D)** Peak **(B)**, steady state at 500 s of monitoring **(C)** and the area under the curve, AUC **(D)** of [Ca^2+^]_c_ transients. Data are mean + SEM for at least six samples from independent experiments. For statistical analysis, one-way ANOVA was used. In **(B–D)**, the means were compared to the G-1–treated mean value. In **(C)**, comparisons with control (black asterisks) and with G-1–treated values (red asterisks) were conducted (**p < 0.01, ***p < 0.001, ****p < 0.0001).

It was demonstrated in several non-lymphoid cellular models that activation of the GPER by natural estrogen or artificial modulators caused rapid non-genomic responses, including Ca^2+^ mobilization (Ariazi et al., 2010; Ren and Wu., 2012; Yang et al., 2017). In the present study, we evaluated the intracellular Ca^2+^ dynamics in response to G-1 administration (1 µM) in Jurkat cells, loaded with Fluo 4. As expected, G-1 elicited a rapid cytosolic Ca^2+^ ([Ca^2+^]_c_) rise, which did not return to the basal level over at least 10 min (Figures 3A–D, red). The observed Ca^2+^ response was efficiently inhibited by pre-incubation with G-36 (Figures 3A–D, blue). Thus, the response was mediated by the GPER. Increase of [Ca^2+^]_c_ may be caused by the influx of Ca^2+^ from the extracellular space or by its release from the intracellular stores. It has been demonstrated previously that G-1 can cause endoplasmic reticulum stress, accompanied by Ca^2+^ release from the endoplasmic reticulum into the cytosol, which, in turn, contributes to the triggering of cell death (Ariazi et al., 2010; Vo et al., 2019). It is also well known that in signaling pathways, triggered by G-protein–coupled receptors, the formation of inositol 1, 4, 5 trisphosphate (IP3) frequently occurs. IP3 binds to the IP3 receptor (IP3R) channel in the endoplasmic reticulum membrane, which, upon this activation, mediates the Ca^2+^ release from the endoplasmic reticulum (Marks, 1997). To test this possibility, a permeable IP3R blocker 2-APB was used (Maruyama et al., 1997). We observed that 2-APB efficiently suppressed the [Ca^2+^]_c_ rise generated by G-1 (Figures 3A–D, orange). Hence, G-1, through the GPER, orchestrates the IP3-dependent Ca^2+^ release from intracellular stores. It is also well known that the Ca^2+^ release from the endoplasmic reticulum causes the activation of store-operated Ca^2+^ channels in the plasma membrane. To estimate the contribution of Ca^2+^ entry from the extracellular space to the observed [Ca^2+^]_c_ rise, the experiments were carried out in a Ca^2+^-free medium (see methods). In contrast to the results obtained in the solutions containing Ca^2+^, under Ca^2+^-free conditions, G-1 provoked a rapid transient [Ca^2+^]_c_ rise followed by a decrease to levels lower than the basal one (Figures 3A–D, green). Summarizing, we demonstrate that G-1, through GPER and IP3R, initially triggered Ca^2+^ release from the endoplasmic reticulum, which consequently activates the store-operated Ca^2+^ entry. Correspondingly, the GPER-mediated Ca^2+^ rise and the functional impact of such Ca^2+^ signals have been recently addressed (DingGao et al., 2019; Vo et al., 2019; Tran 2020).

### G-1 Causes Rapid Generation of ROS

**FIGURE 4:**
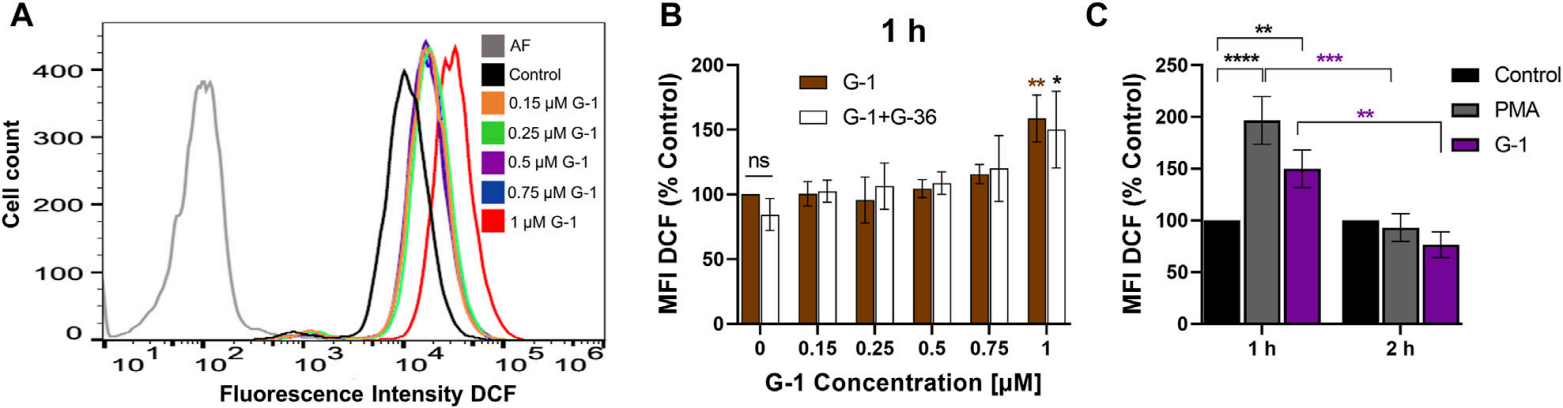
G-1 induces ROS production in Jurkat cells. Cells were treated as indicated during 1 or 2 h and stained with the indicator of ROS activity DCF-DA (5 μM, 30 min). DCF fluorescence was collected by flow cytometry, and mean fluorescence intensity (MFI) was determined. **(A)** Representative histogram obtained with G-1–treated cells. The autofluorescent population (AF) is shown in gray. **(B)** Jurkat cells were pre-incubated with G-36 (10 μM, 30 min), or not, and treated with growing concentrations of G-1 for 1 h. MFI values of treated cells were normalized to control (untreated cells) and shown as mean ± SEM. Comparison of the mean of treated samples to untreated control was performed using two-way ANOVA and a Dunnett *post hoc* test, *p < 0.05, **p < 0.01. Comparison of G-36 pre-incubated with the corresponding not pre-incubated samples was also performed for each G-1 concentration, using two-way ANOVA and Sidak *post hoc* testing; no statistical significance was found. **(C)** MFI DCF values were obtained after 1 and 2 h of incubation with G-1 (1 µM) or PMA (5 µM) as a positive control and normalized to untreated control. Data are mean ± SEM, N ≥ 3. Statistical analysis was performed by comparing the mean of treated and control samples using two-way ANOVA and Tukey *post hoc* analysis (black asterisks); the same treatments after 1 and 2 h were also compared using two-way ANOVA and Sidak *post hoc* analysis (purple asterisks); **p < 0.01, ***p < 0.001, ****p < 0.0001.

ROS production is an important mechanism, involved in cytotoxicity of many anticancer drugs (Kim et al., 2019). Intracellular ROS accumulation was observed in assays with human breast adenocarcinoma and colorectal cancer cells treated with G-1 (Wei et al., 2014; Liu et al., 2017). We evaluated the effect of G-1 on intracellular ROS production by loading the Jurkat cells with the non-fluorescent compound DCFH-DA and measuring the fluorescence intensity of its oxidized (fluorescent) form DCF by flow cytometry (Figures 4A,B). We observed a significant increase in intracellular ROS production within 1 h of G-1 treatment. The same elevated levels of ROS were recorded in cultures pre-incubated with G-36, indicating that the GPER was not involved in the underlying mechanism (Figure 4B). G-36 alone did not stimulate the production of ROS (data not shown). ROS are known to have a very short half-life (Novo and Parola, 2008); then, ROS-related fluorescence decreased to its initial value after 2 h of incubation (Figure 4C).

### G-1 Damages the Mitochondria

**FIGURE 5:**
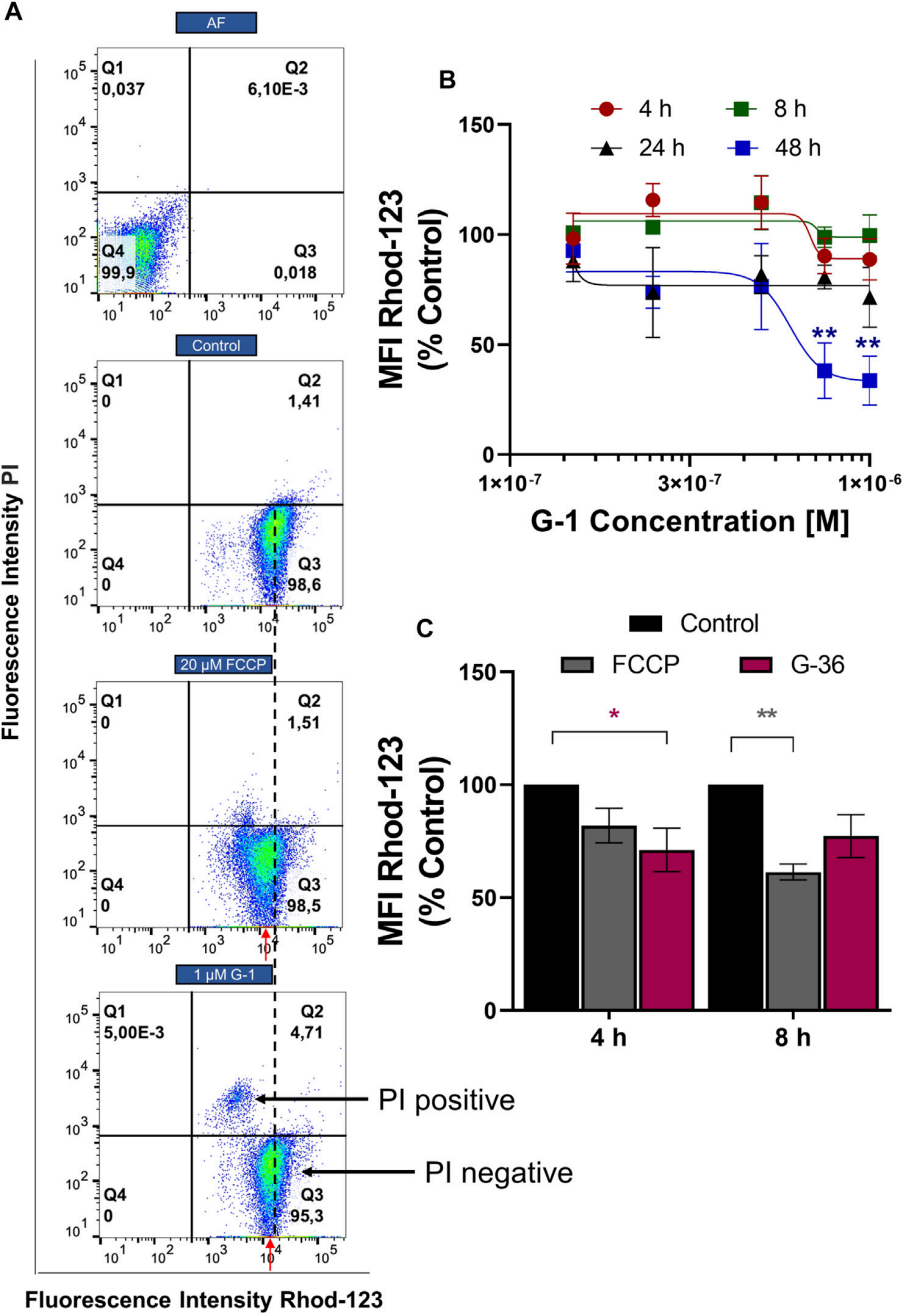
ΔΨm monitoring in Jurkat cells treated with G-1 or G-36. Jurkat cells were treated as indicated for different time intervals, stained with Rhod-123 and PI, and analyzed by flow cytometry. **(A)** Representative dot plots obtained by flow cytometry at 12 h of incubation. FCCP was used as a positive control. The MFI value (Rhod-123) for control population is drawn by a black dotted line. MFI values (Rhod123) for treated populations are indicated by red arrows to demonstrate the shift to the left from the control value. **(B)** MFI mean values (Rhod-123) for PI-negative populations (Q3 as indicated in A) are normalized to control and graphed as a function of G-1 concentrations. Data are mean ± SEM; N ≥ 3. For each time, comparison of control vs. treated samples was performed by one-way ANOVA with Dunnett *post hoc* testing, **p < 0.01. **(C)** Bar charts showing the change in ΔΨm induced by G-36 (10 μM) and FCCP (20 μM). Data are mean ± SEM; N ≥ 3. Two-way ANOVA and a *post hoc* Sidak analysis were performed to compare MFI values (G-36 or FCCP) with corresponding control. *p < 0.05, **p < 0.01.

Mitochondria are important players in neoplastic re-programming of T-ALL. They are involved in the crosstalk between cell bioenergetics, biosynthesis, proliferation, and regulated cell death (Olivas-Aguirre et al., 2019). ΔΨm loss is a classical indicator of mitochondrial damage, and it was observed in tumor cells treated with G-1 (Wei et al., 2014; Liu et al., 2017). ΔΨm changes in Jurkat cells were monitored within 4–48 h of treatment with G-1, using a mitochondrial-specific fluorescent probe Rhod-123 and flow cytometry. Since dead necrotic cells lose ΔΨm independently on the underlying cell death mechanism, they were excluded from the analysis. In other words, cells were co-stained with PI and only PI-negative population, which includes live and early apoptotic cells, was analyzed (Figure 5A, Q3). This approach allows the determination of the initial moment of mitochondrial damage. The protonophore FCCP (20 μM) was used as a positive control. In our experimental conditions, the statistically significant ΔΨm decrease (evaluated by normalized Rhod MFI in PI-negative cells) was observed at 48 h of treatment with 0.75–1 μM G-1 (Figure 5B). Unexpectedly, nontoxic concentration of the GPER antagonist G-36 produced a notable decrease of ΔΨm in Jurkat cells already after 4 h of incubation (Figure 5C). It can be assumed that it was due to this effect on the mitochondria that G-36 potentiated the cytotoxic effect of G-1 in Jurkat cells (Figure 2A).

### Activated CD4^+^ T Cells are Less Sensitive to the G-1 Antiproliferative Effect.

**FIGURE 6:**
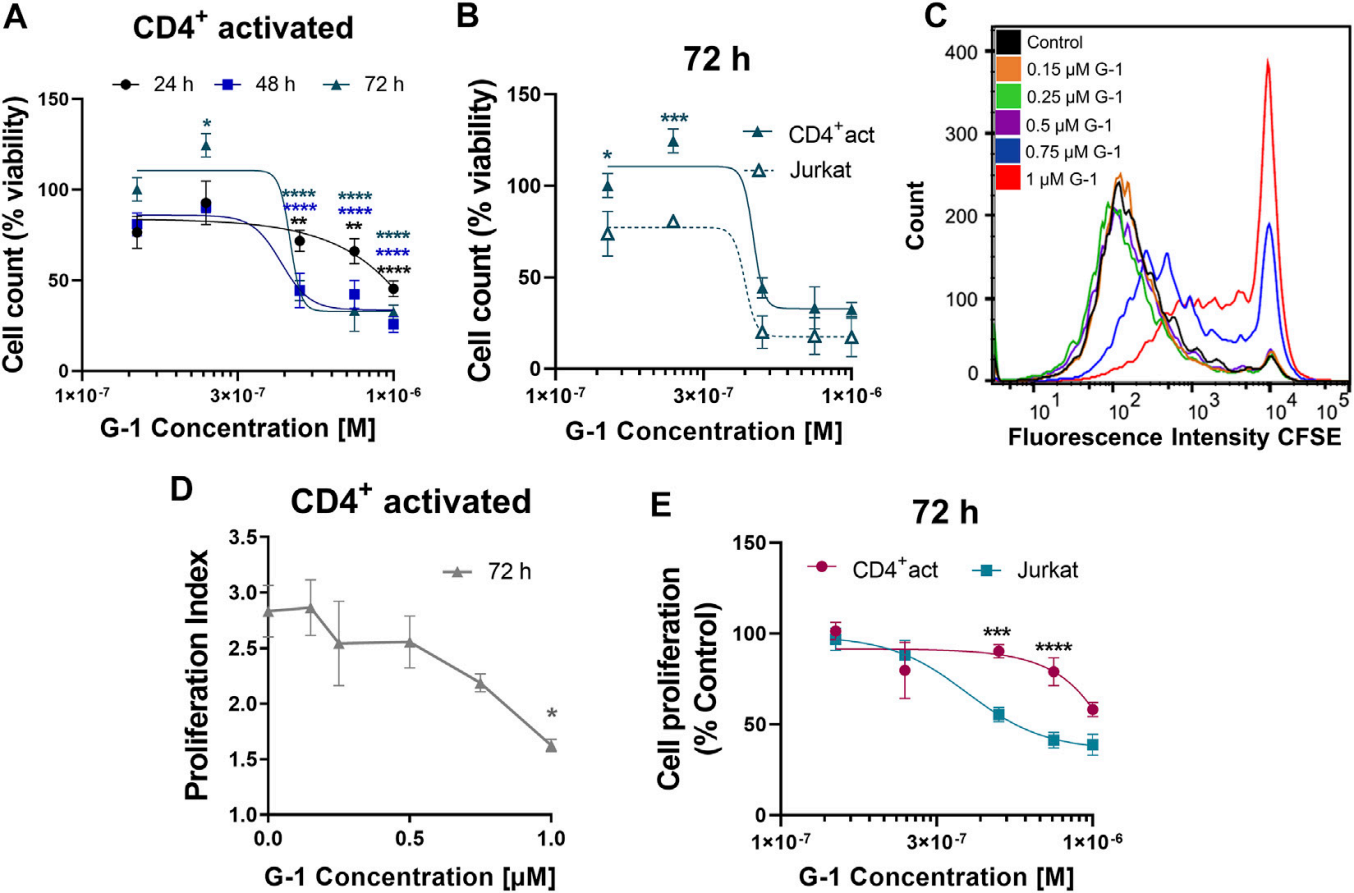
Effect of G-1 on cell viability and proliferation of CD4^+^ lymphocytes. **(A)** Viability of activated non-leukemic CD4^+^ lymphocytes evaluated by cell count (the trypan blue exclusion test) and expressed as a function of G-1 concentration. Data are mean ± SEM. Comparison between control and treated samples was performed by one-way ANOVA with Dunnett *post hoc* testing. N ≥ 3; *p < 0.05, **p < 0.01, ****p < 0.0001. **(B)** Comparison of viability (cell count) non-leukemic CD4^+^ lymphocytes and Jurkat cells treated with different concentrations of G-1 was performed by two-way ANOVA with the Sidak *post hoc* test. Data are mean ± SEM; N ≥ 3; *p < 0.05, ***p < 0.001. **(C)** Representative flow cytometry histogram of CFSE fluorescence intensity measured in activated CD4^+^ lymphocytes treated with different concentrations of G-1 for 72 h. **(D)** Proliferation index expressed as a function of G-1 concentration (FlowJo Software, proliferation tool). Data are mean ± SEM; *N* = 3; *p < 0.05. Statistical significance was obtained by means of comparing each G-1 concentration to their control with a one-way ANOVA and a Dunnett *post hoc* testing. **(E)** Comparison of the decrease in cell proliferation caused by G-1 in CD4^+^ lymphocytes and Jurkat cells. Data are mean ± SEM; ***p < 0.001, ****p < 0.0001. Comparison between the CD4^+^-activated lymphocyte group (pink line) and Jurkat cell line (blue line) was made by two-way ANOVA with Sidak *post hoc* testing.

Non-leukemic CD4^+^ lymphocytes were activated and subsequently treated with G-1 during 24–72 h, and the number of viable cells was estimated every 24 h (Figure 6A). It was demonstrated that non-leukemic lymphocytes presented a lower sensitivity to G-1 than leukemic Jurkat cells (Figure 6B, Supplementary Table S4). Additionally, the classical CFSE-based assay was undertaken, in which CD4^+^ lymphocytes isolated from non-leukemic patients were activated in the presence of increasing concentrations of G-1 over 72 h (Figure 6C). The proliferation rate was evaluated by calculating the proliferation index, which was decreased at high G-1 concentrations (Figure 6D). Comparative analysis revealed that G-1 has a stronger antiproliferative effect on Jurkat cells than on healthy T lymphocytes (Figure 6E, Supplementary Table S4).

## Discussion

The anticancer effect of G-1 was previously demonstrated on tumors of various histogenesis (Supplementary Table S1) but not on T-ALL. In the present work, using leukemic Jurkat and CCRF-CEM cell lines, we have demonstrated that G-1 possesses antileukemic properties. In particular, G-1 suppresses cell proliferation by arresting cell cycle progression in the G2/M phase and induces apoptosis (Figure 1). G-1 sensitivity was slightly different in the two cell lines used, with Jurkat being more sensitive, which may reflect the variability between patients. G-1 is a highly selective GPER agonist, and many research groups have demonstrated that its cytotoxic and anti-proliferative effects are mediated by this receptor in the lungs (Liu et al., 2019), breast (Wei et al., 2014; Weißenborn et al., 2014), gastric (Lee et al., 2019), ovarian (Albanito et al., 2007; Han et al., 2021), and mantle cell lymphoma (Zhou et al., 2021) cell lines. However, there is also evidence that G-1 directly targets and disrupts microtubules in endothelial (Holm et al., 2012), ovarian (Wang et al., 2013), and breast cancer (Lv et al., 2017) cells. An essential role of microtubules in many cellular processes such as motility, intracellular trafficking, cell growth, and division is widely recognized. Importantly, the functions of microtubules are crucially dependent on their precisely regulated polymerization dynamics (Mukhtar et al., 2014). The direct suppressive effect of G-1 on the assembly of microtubules was demonstrated using the *in vitro* microtubule assembly test (Lv et al., 2017). More precisely, it was shown that G-1 attaches to the colchicine binding site on tubulin, preventing its polymerization and subsequent assembly of the mitotic spindle. As a result, cells are arrested in the G2 phase and early mitosis (Wang et al., 2013; Lv et al., 2017). Here, we present evidence for similar mechanisms of the G-1 action on Jurkat cells, where antiproliferative and damaging effects were reached at the narrow range of G-1 concentrations (0.25–1 µM) (Figures 1A–C,I,J). Highly cooperative G-1 effects seem to be associated with a disturbed dynamics and damage of microtubules, manifested from 0.5 µM (Figure 2D). Remarkably, cytoskeleton disorders, cell cycle arrest, and apoptosis were not prevented by a specific GPER antagonist G-36 (Figures 2A–C,E; Supplementary Figures S3F–K).

GPER belongs to the superfamily of seven transmembrane receptors (7TMR). Activation of 7TMR often leads to a Ca^2+^ response (Gudermann and Bader, 2015). Such an effect has been observed when activating the GPER (Revankar et al., 2005; Ariazi et al., 2010; Dennis et al., 2011; Luo et al., 2014; Ding et al., 2019; Vo et al., 2019; Tran 2020). In our experiments, G-1 caused a rapid increase in [Ca^2+^]_c_, which was initially mediated by the Ca^2+^ release from the endoplasmic reticulum through the IP3R channel, followed by a prolonged influx of Ca^2+^ from the extracellular space (Figure 3), most likely due to the activation of so named store-operated calcium entry (SOCE), which is the main Ca^2+^ entry channel in lymphocytes (Zweifach and Lewis, 1993). Notably, G-36 effectively prevented this G-1-mediated Ca^2+^ response (Figure 3), pointing out that this effect is triggered through the GPER. Hence, it is unlikely that this early [Ca ^2+^]_c_ rise is related to the cytotoxic effects of G-1, which were independent of the GPER (Figure 2). However, considering that mitochondria acts as the main regulator of intracellular Ca^2+^, we cannot rule out that the prolonged Ca^2+^ entry influences the mitochondrial energetic or redox status.

Chemical compounds targeting microtubules are an important strategy in chemotherapeutic protocols. Due to the high toxicity and undesirable side effects of the currently used drugs, the search for new tubulin polymerization inhibitors, both natural and synthetic, continues (Kaur et al., 2014). Microtubule-destabilizing agents such as vinca alkaloids, including vincristine, vinblastine, and vinorelbine, are often used in chemotherapeutic protocols for hematological malignancies (Stanton et al., 2011). G-1 can be suggested as a possible alternative in chemotherapeutic protocols for T-ALL treatment, although additional experiments are still needed to identify the benefits of its use. To date, *in vivo* studies in xenograft mouse models have shown that the concentrations of G-1, which effectively suppress the growth of breast and ovarian cancer, do not affect the body weight, social behavior, and reproductive physiology, indicating a relatively low toxicity of G-1 for healthy tissues (Lv et al., 2017). Notably, T-ALL leukemic cells are quite sensitive to G-1, when compared to other types of cancers (Supplementary Table S1), and are also more sensitive than healthy T lymphocytes.

Finally, the phase 1 clinical trial (NCT04130516) is currently in progress to assess the safety, tolerability, pharmacokinetics, and antitumor effects of LNS8801 (SRR G-1, Linnaeus Therapeutics Inc.) in patients with advanced or recurrent treatment-refractory solid malignancies, upon oral administration. Very encouraging data from these studies, which demonstrated good tolerability and antitumor activity of LNS8801 both as monotherapy or in combination with pembrolizumab, have been reported recently (Muller et al., 2021). Since G-1 shows antileukemic activity in pre-clinical studies and LNS8801 (SRR G-1) is well tolerated in patients, T-ALL may, in the near future, be recommended to the list of malignancies for clinical trials with this compound.

## Data Availability

The original contributions presented in the study are included in the article/Supplementary Material; further inquiries can be directed to the corresponding author.

## Supplementary Material

Supplementary Figure S1 Subcellular localization of GPER in breast cancer and T leukemic cell lines. Confocal micrographs showing localization of GPER (red), Na^+^/K^+^ ATPase (green), and their merged images. MCF-7 and MDA-MB-231 breast cancer cell lines were included in the panel for comparison. Na^+^/K^+^ ATPase localization was used for reference. Images taken with 40x objective and edited with ZEN lite software 3.0. Nucleus: DAPI. Scale bar: 20 μm.

Supplementary Figure S2 Cytotoxic effect of G-1 in two different RPMI media. Jurkat cells were cultured in RPMI medium without phenol red and supplemented with 10% FCS dialyzed and treated with growing concentrations of G-1 for the indicated periods of time. **(A)** Cell viability evaluated by live cell count (trypan blue exclusion test) at indicated time points. Data (normalized to 0 h time point) are mean ± SEM; N ≥ 3; **p* < 0.05, ****p* < 0.001, *****p* < 0.0001; one-way ANOVA with Dunnett post hoc testing. **(B–D)** Cell count of Jurkat cells treated with different concentrations of G-1 for 24 h **(B)**, 48 h **(C)** and 72 h **(D)** cultured in Advanced RPMI medium with phenol red and supplemented with 5% FBS (red lines), and RPMI medium without phenol red and supplemented with 10% FCS dialyzed (black lines). Data are mean ± SEM; N ≥ 3. Comparison was done by two-way ANOVA with Sidak post hoc test; no statistically significant difference was obtained. **(E,F)** Jurkat cells were incubated without treatment (control) and in presence of G-1 (0.15–1 µM) for 24 h. Cells were fixed, stained with PI and processed for flow cytometry. **(E)** Representative ModFit histograms of untreated cells and cells incubated with G-1 (1 µM, 24 h). G0/G1 peak is pink, G2/M peak is purple, S phase and subG1 damaged population is light-purple. **(F)** Bar charts showing the percentage of the Jurkat cells in different subpopulations corresponding to G1, S and G2 phases at 24 h of G-1 treatment. **(G)** Jurkat cells treated with G-1 for 24 h. Bar charts showing percentage of cells in Q1 necrotic (Annexin V/AlexaFluor488^-^PI^+^); Q2 late apoptotic/necrotic (Annexin V/AlexaFluor488^+^PI^+^); Q3 apoptotic (Annexin V/AlexaFluor488^+^PI^-^); Q4 viable (Annexin V/AlexaFluor488^-^PI^-^). Data are shown as mean ± SEM; N ≥ 3; *****p* < 0.0001. Comparison between control (without treatment) and G-1 - treated samples was made by two-way ANOVA with Dunnett post hoc testing. **(H,I)** Percentage of viable cells (Annexin V/AlexaFluor488^-^PI^-^ cells) **(H)** and apoptotic cells (Annexin V/AlexaFluor488^+^PI^-^) **(I)** at 24 h of treatment. Data are mean ± SEM; N ≥ 3; Comparison between Jurkat cells cultured in Advanced RPMI medium with phenol red and RPMI medium without phenol red was performed by two-way ANOVA with Sidak post hoc testing. No statistical difference was revealed.

Supplementary Figure S3 Effect of G-1 + G-36 in Jurkat and CCRF-CEM cell lines. **(A–C)** Bar charts show cell count (trypan blue exclusion test) of Jurkat **(A,B)** and CCRF-CEM **(C)** cells treated with G-36 10 µM for 4–72 h. Data are normalized to control. **(D,E)** Bar charts showing the mean fluorescence peak (MFI) of CFSE in Jurkat **(D)** and CCRF-CEM **(E)** cells treated with G-36 10 µM for 24–72 h. Data are normalized to control. **(A–E)** Comparison between G-36 and the control of its corresponding time was made by two-way ANOVA with Sidak post hoc testing. **(F–I)** Jurkat **(F–G)** and CCRF-CEM **(H,I)** cells were pre-incubated with G-36 (10 µM, 30 min), seeded in Advanced RPMI medium with different concentrations of G-1 and cultivated during indicated periods of time. Cell viability **(F,H)** and cell proliferation **(G,I)** was evaluated and graphed as function of G-1 concentration. **(J)** Jurkat cells cultured in RPMI medium without phenol red and supplemented with 10% FCS dialyzed were treated with G-1 (0.15–10 µM) + G-36 (10 µM, pre-incubated) for 24–72 h. **(F–J)** Data are normalized to control and shown as mean ± SEM; N ≥ 3. Comparison between G-1 and G1+G-36 was performed by two-way ANOVA with Sidak post hoc testing. **(K)** Jurkat cells cultured in Advanced RPMI medium were treated with growing concentrations of G-1 for 24 h. Bar charts showing percentage of different gates obtained by flow cytometric corresponding to necrotic (Q1), late apoptotic/necrotic (Q2), apoptotic (Q3) and viable cells (Q4). Data are shown as mean ± SEM; N ≥ 3; *****p* < 0.0001. Comparison between control (without treatment) and samples treated with G-1 + G-36 was made by two-way ANOVA with Dunnett post hoc testing.

## Abbreviations

ALL, acute lymphoblastic leukemia; ANOVA, analysis of variance; ATCC, American Type Culture Collection; CFSE, carboxy-fluorescein succinimidyl ester; CTCF, corrected total cell fluorescence; DCF, 2′,7′dichlorofluorescin diacetate; FBS, fetal bovine serum; FCCP, carbonyl cyanide 4 (trifluoromethoxy) phenylhydrazone; FCS, fetal calf serum; GPER, G-protein–coupled estrogen receptor(s); MFI, median fluorescence intensity; PBMC, peripheral blood mononuclear cells; PI, propidium iodide; PMA, phorbol myristate acetate; Rhod-123, rhodamine-123; ROS, reactive oxygen species; SEM, standard error of mean; T-ALL, acute lymphoblastic leukemia of T lineage; TCR, T-cell receptor; ΔΨm, mitochondrial membrane potential.

## References

[B1] Albanito L. Madeo A. Lappano R. Vivacqua A. Rago V. Carpino A. (2007). G Protein-Coupled Receptor 30 (GPR30) Mediates Gene Expression Changes and Growth Response to 17β-Estradiol and Selective GPR30 Ligand G-1 in Ovarian Cancer Cells. Cancer Res. 67 (4), 1859–1866. 10.1158/0008-5472.CAN-06-2909 17308128

[B2] Altmann J. B. Yan G. Meeks J. F. Abood M. E. Brailoiu E. Brailoiu G. C. (2015). G Protein-Coupled Estrogen Receptor-Mediated Effects on Cytosolic Calcium and Nanomechanics in Brain Microvascular Endothelial Cells. J. Neurochem. 133 (5), 629–639. 10.1111/jnc.13066 25703621 PMC4562690

[B3] Ariazi E. A. Brailoiu E. Yerrum S. Shupp H. A. Slifker M. J. Cunliffe H. E. (2010). The G Protein-Coupled Receptor GPR30 Inhibits Proliferation of Estrogen Receptor-Positive Breast Cancer Cells. Cancer Res. 70 (3), 1184–1194. 10.1158/0008-5472.CAN-09-3068 20086172 PMC2879282

[B4] Bai L.-Y. Weng J.-R. Hu J.-L. Wang D. Sargeant A. M. Chiu C.-F. (2013). G15, a GPR30 Antagonist, Induces Apoptosis and Autophagy in Human Oral Squamous Carcinoma Cells. Chemico-Biological Interactions 206 (2), 375–384. 10.1016/j.cbi.2013.10.014 24161432

[B5] Berthois Y. Katzenellenbogen J. A. Katzenellenbogen B. S. (1986). Phenol Red in Tissue Culture media Is a Weak Estrogen: Implications Concerning the Study of Estrogen-Responsive Cells in Culture. Proc. Natl. Acad. Sci. 83 (8), 2496–2500. 10.1073/pnas.83.8.2496 3458212 PMC323325

[B6] Bologa C. G. Revankar C. M. Young S. M. Edwards B. S. Arterburn J. B. Kiselyov A. S. (2006). Virtual and Biomolecular Screening Converge on a Selective Agonist for GPR30. Nat. Chem. Biol. 2 (4), 207–212. 10.1038/nchembio775 16520733

[B7] Brailoiu G. C. Arterburn J. B. Oprea T. I. Chitravanshi V. C. Brailoiu E. (2013). Bradycardic Effects Mediated by Activation of G Protein-Coupled Estrogen Receptor in Rat Nucleus Ambiguus. Exp. Physiol. 98 (2), 679–691. 10.1113/expphysiol.2012.069377 23104934 PMC3578005

[B9] Catusse J. Wollner S. Leick M. Schröttner P. Schraufstätter I. Burger M. (2010). Attenuation of CXCR4 Responses by CCL18 in Acute Lymphocytic Leukemia B Cells. J. Cel. Physiol. 225 (3), 792–800. 10.1002/jcp.22284 20568229

[B10] Chimento A. Sirianni R. Casaburi I. Zolea F. Rizza P. Avena P. (2015). GPER Agonist G-1 Decreases Adrenocortical Carcinoma (ACC) Cell Growthin Vitroandin Vivo. Oncotarget 6 (22), 19190–19203. 10.18632/oncotarget.4241 26131713 PMC4662484

[B11] Cirillo F. Pellegrino M. Malivindi R. Rago V. Avino S. Muto L. GPER Is Involved in the Regulation of the Estrogen-Metabolizing CYP1B1 Enzyme in Breast Cancer. Oncotarget (2017) 8(63):106608–106624. 10.18632/oncotarget.22541 29290975 PMC5739760

[B12] Dennis M. K. Burai R. Ramesh C. Petrie W. K. Alcon S. N. Nayak T. K. (2009). *In Vivo* effects of a GPR30 Antagonist. Nat. Chem. Biol. 5 (6), 421–427. 10.1038/nchembio.168 19430488 PMC2864230

[B13] Dennis M. K. Field A. S. Burai R. Ramesh C. Petrie W. K. Bologa C. G. (2011). Identification of a GPER/GPR30 Antagonist with Improved Estrogen Receptor Counterselectivity. J. Steroid Biochem. Mol. Biol. 127 (3–5), 358–366. 10.1016/j.jsbmb.2011.07.002 21782022 PMC3220788

[B14] Ding X. Gao T. Gao T. Gao P. Meng Y. Zheng Y. (2019). Activation of the G Protein-Coupled Estrogen Receptor Elicits Store Calcium Release and Phosphorylation of the Mu-Opioid Receptors in the Human Neuroblastoma SH-Sy5y Cells. Front. Neurosci. 13, 1351. 10.3389/fnins.2019.01351 31920512 PMC6928052

[B15] Filardo E. J. Graeber C. T. Quinn J. A. Resnick M. B. Giri D. DeLellis R. A. (2006). Distribution of GPR30, a Seven Membrane-Spanning Estrogen Receptor, in Primary Breast Cancer and its Association with Clinicopathologic Determinants of Tumor Progression. Clin. Cancer Res. 12 (21), 6359–6366. 10.1158/1078-0432.CCR-06-0860 17085646

[B16] Filardo E. J. Quinn J. A. Bland K. I. Frackelton A. R. (2000). Estrogen-induced Activation of Erk-1 and Erk-2 Requires the G Protein-Coupled Receptor Homolog, GPR30, and Occurs via Trans-activation of the Epidermal Growth Factor Receptor through Release of HB-EGF. Mol. Endocrinol. 14 (10), 1649–1660. 10.1210/mend.14.10.0532 11043579

[B17] Girgert R. Emons G. Gründker C. (2019). Estrogen Signaling in ERα-Negative Breast Cancer: ERβ and GPER. Front. Endocrinol. 9 (871), 1–12. 10.3389/fendo.2018.00781 PMC6333678 30687231

[B18] Gudermann T. Bader M. (2015). Receptors, G Proteins, and Integration of Calcium Signalling. J. Mol. Med. 93, 937–940. 10.1007/s00109-015-1330-y 26293356

[B19] Gui Y. Shi Z. Wang Z. Li J.-J. Xu C. Tian R. (2015). The GPER Agonist G-1 Induces Mitotic Arrest and Apoptosis in Human Vascular Smooth Muscle Cells Independent of GPER. J. Cel. Physiol. 230 (4), 885–895. 10.1002/jcp.24817 25204801

[B20] Han N. Heublein S. Jeschke U. Kuhn C. Hester A. Czogalla B. (2021). The G-Protein-Coupled Estrogen Receptor (GPER) Regulates Trimethylation of Histone H3 at Lysine 4 and Represses Migration and Proliferation of Ovarian Cancer Cells *In Vitro* . Cells 10 (3), 619–642. 10.3390/cells10030619 33799631 PMC8001910

[B21] Hasni M. S. Yakimchuk K. (2019). Expression and Effects of Ligand-Activated Estrogen Receptors in Chronic Lymphocytic Leukemia. Anticancer Res. 39 (1), 167–172. 10.21873/anticanres.13093 30591454

[B22] Holm A. Grände P.-O. Ludueña R. F. Olde B. Prasad V. Leeb-Lundberg L. M. F. (2012). The G Protein-Coupled Oestrogen Receptor 1 Agonist G-1 Disrupts Endothelial Cell Microtubule Structure in a Receptor-independent Manner. Mol. Cel Biochem 366 (1–2), 239–249. 10.1007/s11010-012-1301-3 22451019

[B23] Hsu L.-H. Chu N.-M. Lin Y.-F. Kao S.-H. (2019). G-protein Coupled Estrogen Receptor in Breast Cancer. Ijms 20 (2), 306–322. 10.3390/ijms20020306 PMC6359026 30646517

[B25] Imesch P. Samartzis E. P. Dedes K. J. Fink D. Fedier A. (2013). Histone Deacetylase Inhibitors Down-Regulate G-Protein-Coupled Estrogen Receptor and the GPER-Antagonist G-15 Inhibits Proliferation in Endometriotic Cells. Fertil. Sterility 100 (3), 770–776. 10.1016/j.fertnstert.2013.05.008 23755949

[B26] Jala V. R. Radde B. N. Haribabu B. Klinge C. M. (2012). Enhanced Expression of G-Protein Coupled Estrogen Receptor (GPER/GPR30) in Lung Cancer. BMC Cancer 12 (1), 264–276. 10.1186/1471-2407-12-624 23273253 PMC3557142

[B27] Jenkins J. K. Suwannaroj S. Elbourne K. B. Ndebele K. McMurray R. W. (2001). 17-β-Estradiol Alters Jurkat Lymphocyte Cell Cycling and Induces Apoptosis through Suppression of Bcl-2 and Cyclin A. Int. Immunopharmacology 1 (11), 1897–1911. 10.1016/S1567-5769(01)00114-X 11606022

[B28] Jung J. (2019). Role of G Protein-Coupled Estrogen Receptor in Cancer Progression. ToxicolRes 35 (3), 209–214. 10.5487/TR.2019.35.3.209 PMC6629442 31341549

[B29] Kasioulis I. Das R. M. Storey K. G. (2017). Inter-dependent Apical Microtubule and Actin Dynamics Orchestrate Centrosome Retention and Neuronal Delamination. ELife 6, e26215. 10.7554/eLife.26215 29058679 PMC5653239

[B30] Kaur R. Kaur G. Gill R. K. Soni R. Bariwal J. (2014). Recent Developments in Tubulin Polymerization Inhibitors: An Overview. Eur. J. Med. Chem. 87, 89–124. 10.1016/j.ejmech.2014.09.051 25240869

[B31] Khan D. Cowan C. Ahmed S. A. (2012). Estrogen and Signaling in the Cells of Immune System. Adv. Neuroimmune Biol. 3 (1), 73–93. 10.3233/NIB-2012-012039

[B32] Kim S. J. Kim H. S. Seo Y. R. (2019). Understanding of ROS-Inducing Strategy in Anticancer Therapy. Oxidative Med. Cell Longevity 2019, 1–12. 10.1155/2019/5381692 PMC6939418 31929855

[B33] Kovats S. (2015). Estrogen Receptors Regulate Innate Immune Cells and Signaling Pathways. Cell Immunol. 294 (2), 63–69. 10.1016/j.cellimm.2015.01.018 25682174 PMC4380804

[B34] Kurt A. H. Çelik A. Kelleci B. M. (2015). Oxidative/antioxidative Enzyme-Mediated Antiproliferative and Proapoptotic Effects of the GPER1 Agonist G-1 on Lung Cancer Cells. Oncol. Lett. 10 (5), 3177–3182. 10.3892/ol.2015.3711 26722308 PMC4665271

[B35] Ladikou E.-E. Kassi E. (2017). The Emerging Role of Estrogen in B Cell Malignancies. Leuk. Lymphoma 58 (3), 528–539. 10.1080/10428194.2016.1213828 27557932

[B36] Lappano R. Mallet C. Rizzuti B. Grande F. Galli G. Byrne C. (2019). The Peptide ERα17p Is a GPER Inverse Agonist that Exerts Antiproliferative Effects in Breast Cancer Cells. Cells 8 (6), 590. 10.3390/cells8060590 PMC6627388 31207943

[B37] Lee S.-J. Kim T. W. Park G. L. Hwang Y. S. Cho H. J. Kim J.-T. (2019). G Protein-Coupled Estrogen Receptor-1 Agonist Induces Chemotherapeutic Effect via ER Stress Signaling in Gastric Cancer. BMB Rep. 52, 647–652. 10.5483/BMBRep.2019.52.11.007 31234952 PMC6889890

[B38] Liu C. Liao Y. Fan S. Fu X. Xiong J. Zhou S. (2019). G-protein-coupled Estrogen Receptor Antagonist G15 Decreases Estrogen-Induced Development of Non-small Cell Lung Cancer. Oncol. Res. 27 (3), 283–292. 10.3727/096504017X150357959046710.3727/096504017x15035795904677 28877783 PMC7848463

[B39] Liu Q. Chen Z. Jiang G. Zhou Y. Yang X. Huang H. (2017). Epigenetic Down Regulation of G Protein-Coupled Estrogen Receptor (GPER) Functions as a Tumor Suppressor in Colorectal Cancer. Mol. Cancer 16 (1), 1–14. 10.1186/s12943-017-0654-3 28476123 PMC5418684

[B40] Luo H. Yang G. Yu T. Luo S. Wu C. Sun Y. (2014). GPER-mediated Proliferation and Estradiol Production in Breast Cancer-Associated Fibroblasts. Endocr. Relat. Cancer 21 (2), 355–369. 10.1530/ERC-13-0237 24481325 PMC3959763

[B41] Lv X. Wang C. (2014). G-1: New Potential Therapeutic Option for Ovarian Cancer. Cancer Cell Microenviron 1, e27. 10.14800/ccm.27

[B42] Lv X. He C. Huang C. Hua G. Wang Z. Remmenga S. W. (2017). G-1 Inhibits Breast Cancer Cell Growth via Targeting Colchicine-Binding Site of Tubulin to Interfere with Microtubule Assembly. Mol. Cancer Ther. 16, 1080–1091. 10.1158/1535-7163.MCT-16-0626 28258163 PMC5457708

[B43] Marks A. R. (1997). Intracellular Calcium-Release Channels: Regulators of Cell Life and Death. Am. J. Physiol. 272 (2Pt2), H597–H605. 10.1152/ajpheart.1997.272.2.H5910.1152/ajpheart.1997.272.2.H597 9124414

[B44] Maruyama T. Kanaji T. Nakade S. Kanno T. Mikoshiba K. (1997). 2APB, 2-Aminoethoxydiphenyl Borate, a Membrane-Penetrable Modulator of Ins(1,4,5)P3-Induced Ca2+ Release. J. Biochem. 122 (3), 498–505. 10.1093/oxfordjournals.jbchem.a021780 9348075

[B45] Moreno-Ulloa A. Miranda-Cervantes A. Licea-Navarro A. Mansour C. Beltrán-Partida E. Donis-Maturano L. (2018). (-)-Epicatechin Stimulates Mitochondrial Biogenesis and Cell Growth in C2C12 Myotubes via the G-Protein Coupled Estrogen Receptor. Eur. J. Pharmacol. 822, 95–107. 10.1016/j.ejphar.2018.01.014 29355558 PMC5809192

[B46] Mori T. Ito F. Matsushima H. Takaoka O. Tanaka Y. Koshiba A. (2015). G Protein-Coupled Estrogen Receptor 1 Agonist G-1 Induces Cell Cycle Arrest in the Mitotic Phase, Leading to Apoptosis in Endometriosis. Fertil. Sterility 103 (5), 1228–1235. 10.1016/j.fertnstert.2015.01.026 25724739

[B47] Mukhtar E. Adhami V. M. Mukhtar H. (2014). Targeting Microtubules by Natural Agents for Cancer Therapy. Mol. Cancer Ther. 13 (2), 275–284. 10.1158/1535-7163.MCT-13-0791 24435445 PMC3946048

[B48] Muller C. Brown-Glaberman U. A. Chaney M. F. Garyantes T. LoRusso P. McQuade J. L. (2021). Phase 1 Trial of a Novel, First-In-Class G Protein-Coupled Estrogen Receptor (GPER) Agonist, LNS8801, in Patients with Advanced or Recurrent Treatment-Refractory Solid Malignancies. Jco 39 (15Suppl. l), 3084. 10.1200/JCO.2021.39.15_suppl.3084

[B49] Natale C. A. Li J. Pitarresi J. R. Norgard R. J. Dentchev T. Capell B. C. (2020). Pharmacologic Activation of the G Protein-Coupled Estrogen Receptor Inhibits Pancreatic Ductal Adenocarcinoma. Cell Mol. Gastroenterol. Hepatol. 10 (4), 868–880. 10.1016/j.jcmgh.2020.04.016 32376419 PMC7578406

[B50] Nayak T. K. Dennis M. K. Ramesh C. Burai R. Atcher R. W. Sklar L. A. (2010). Influence of Charge on Cell Permeability and Tumor Imaging of GPR30-Targeted 111In-Labeled Nonsteroidal Imaging Agents. ACS Chem. Biol. 5 (7), 681–690. 10.1021/cb1000636 20486699 PMC2912446

[B51] Novo E. Parola M. (2008). Redox Mechanisms in Hepatic Chronic Wound Healing and Fibrogenesis. Fibrogenesis Tissue Repair 1 (1), 5. 10.1186/1755-1536-1-5 19014652 PMC2584013

[B52] Olivas‐Aguirre M. Pottosin I. Dobrovinskaya O. (2019). Mitochondria as Emerging Targets for Therapies against T Cell Acute Lymphoblastic Leukemia. J. Leukoc. Biol. 105 (5), 935–946. 10.1002/JLB.5VMR0818-330RR 30698851

[B53] Prossnitz E. R. Barton M. (2011). The G-Protein-Coupled Estrogen Receptor GPER in Health and Disease. Nat. Rev. Endocrinol. 7 (12), 715–726. 10.1038/nrendo.2011.122 21844907 PMC3474542

[B54] Quah B. J. C. Warren H. S. Parish C. R. (2007). Monitoring Lymphocyte Proliferation *In Vitro* and *In Vivo* with the Intracellular Fluorescent Dye Carboxyfluorescein Diacetate Succinimidyl Ester. Nat. Protoc. 2 (9), 2049–2056. 10.1038/nprot.2007.296 17853860

[B55] Raetz E. A. Teachey D. T. (2016). T-cell Acute Lymphoblastic Leukemia. Hematology 2016 (1), 580–588. 10.1182/asheducation-2016.1.580 27913532 PMC6142501

[B56] Ren J. Wu J. H. (2012). 17β-Estradiol Rapidly Activates Calcium Release from Intracellular Stores via the GPR30 Pathway and MAPK Phosphorylation in Osteocyte-like MLO-Y4 Cells. Calcif Tissue Int. 90 (5), 411–419. 10.1007/s00223-012-9581-x 22392527

[B57] Revankar C. M. Cimino D. F. Sklar L. A. Arterburn J. B. Prossnitz E. R. (2005). A Transmembrane Intracellular Estrogen Receptor Mediates Rapid Cell Signaling. Science 307 (5715), 1625–1630. 10.1126/science.1106943 15705806

[B58] Ribeiro M. P. C. Santos A. E. Custódio J. B. A. (2017). The Activation of the G Protein-Coupled Estrogen Receptor (GPER) Inhibits the Proliferation of Mouse Melanoma K1735-M2 Cells. Chemico-Biological Interactions 277, 176–184. 10.1016/j.cbi.2017.09.017 28947257

[B59] Rudelius M. Rauert-Wunderlich H. Hartmann E. Hoster E. Dreyling M. Klapper W. (2015). The G Protein-Coupled Estrogen Receptor 1 (GPER-1) Contributes to the Proliferation and Survival of Mantle Cell Lymphoma Cells. Haematologica 100, e458–e461. 10.3324/haematol.2015.127399 26250574 PMC4825299

[B60] Sánchez-Aguilera A. Méndez-Ferrer S. (2016). Regulation of Hematopoietic Progenitors by Estrogens as a Basis for New Antileukemic Strategies. Mol. Cell Oncol. 3 (1), e1009728. 10.1080/23723556.2015.1009728 27308525 PMC4845161

[B61] Shakya S. Sharma P. Bhatt A. M. Jani R. A. Delevoye C. Gangi Setty S. R. (2018). Rab22A Recruits BLOC ‐1 and BLOC ‐2 to Promote the Biogenesis of Recycling Endosomes. EMBO Rep. 19 (12), 1–17. 10.15252/embr.201845918 30404817 PMC6280653

[B62] Sjöström M. Hartman L. Grabau D. Fornander T. Malmström P. Nordenskjöld B. (2014). Lack of G Protein-Coupled Estrogen Receptor (GPER) in the Plasma Membrane Is Associated with Excellent Long-Term Prognosis in Breast Cancer. Breast Cancer Res. Treat. 145 (1), 61–71. 10.1007/s10549-014-2936-4 24715381

[B63] Stanton R. A. Gernert K. M. Nettles J. H. Aneja R. (2011). Drugs that Target Dynamic Microtubules: A New Molecular Perspective. Med. Res. Rev. 31, 443–481. 10.1002/med.20242 21381049 PMC3155728

[B64] Torres‐López L. Maycotte P. Liñán‐Rico A. Liñán‐Rico L. Donis‐Maturano L. Delgado‐Enciso I. (2019). Tamoxifen Induces Toxicity, Causes Autophagy, and Partially Reverses Dexamethasone Resistance in Jurkat T Cells. J. Leukoc. Biol. 105 (5), 983–998. 10.1002/JLB.2VMA0818-328R 30645008

[B65] Tran Q.-K. (2020). Reciprocality between Estrogen Biology and Calcium Signaling in the Cardiovascular System. Front. Endocrinol. 11, 568203. 10.3389/fendo.2020.568203 PMC7550652 33133016

[B66] Tutzauer J. Sjöström M. Bendahl P.-O. Rydén L. Fernö M. Leeb-Lundberg L. M. F. (2020). Plasma Membrane Expression of G Protein-Coupled Estrogen Receptor (GPER)/G Protein-Coupled Receptor 30 (GPR30) Is Associated with Worse Outcome in Metachronous Contralateral Breast Cancer. PLoS One 15 (4), e0231786. 10.1371/journal.pone.0231786 32302351 PMC7164601

[B67] Vadillo E. Dorantes-Acosta E. Pelayo R. Schnoor M. (2018). T Cell Acute Lymphoblastic Leukemia (T-ALL): New Insights into the Cellular Origins and Infiltration Mechanisms Common and Unique Among Hematologic Malignancies. Blood Rev. 32 (1), 36–51. 10.1016/j.blre.2017.08.006 28830639

[B68] Vo D.-K. H. Hartig R. Weinert S. Haybaeck J. Nass N. (2019). G-protein-coupled Estrogen Receptor (GPER)-specific Agonist G1 Induces ER Stress Leading to Cell Death in MCF-7 Cells. Biomolecules 9 (9), 503–521. 10.3390/biom9090503 PMC6769846 31540491

[B69] Wang C. Lv X. Jiang C. Davis J. S. (2012). The Putative G-Protein Coupled Estrogen Receptor Agonist G-1 Suppresses Proliferation of Ovarian and Breast Cancer Cells in a GPER-independent Manner. Am. J. Transl Res. 4 (4), 390–402. 23145207 PMC3493027

[B70] Wang C. Lv X. He C. Hua G. Tsai M.-Y. Davis J. S. (2013). The G-Protein-Coupled Estrogen Receptor Agonist G-1 Suppresses Proliferation of Ovarian Cancer Cells by Blocking Tubulin Polymerization. Cell Death Dis 4 (10), e869. 10.1038/cddis.2013.397 24136233 PMC3920961

[B71] Wei W. Chen Z.-J. Zhang K.-S. Yang X.-L. Wu Y.-M. Chen X.-H. (2014). The Activation of G Protein-Coupled Receptor 30 (GPR30) Inhibits Proliferation of Estrogen Receptor-Negative Breast Cancer Cells *In Vitro* and *In Vivo* . Cel Death Dis 5 (10), e1428. 10.1038/cddis.2014.398 PMC4649509 25275589

[B72] Weißenborn C. Ignatov T. Ochel H.-J. Costa S. D. Zenclussen A. C. Ignatova Z. (2014). GPER Functions as a Tumor Suppressor in Triple-Negative Breast Cancer Cells. J. Cancer Res. Clin. Oncol. 140 (5), 713–723. 10.1007/s00432-014-1620-8 24553912 PMC11823775

[B73] Xu S. Yu S. Dong D. Lee L. T. O. (2019). G Protein-Coupled Estrogen Receptor: A Potential Therapeutic Target in Cancer. Front. Endocrinol. 10, 725. 10.3389/fendo.2019.00725 PMC6823181 31708873

[B74] Yakimchuk K. Jondal M. Okret S. (2013). Estrogen Receptor α and β in the normal Immune System and in Lymphoid Malignancies. Mol. Cell Endocrinol. 375 (1–2), 121–129. 10.1016/j.mce.2013.05.016 23707618

[B75] Yang D.-L. Xu J.-W. Zhu J.-G. Zhang Y.-L. Xu J.-B. Sun Q. (2017). Role of GPR30 in Estrogen-Induced Prostate Epithelial Apoptosis and Benign Prostatic Hyperplasia. Biochem. Biophysical Res. Commun. 487 (3), 517–524. 10.1016/j.bbrc.2017.04.047 28412354

[B76] Yedjou C. Cameron J. Mbemi A. T. Tchounwou P. (2015). β-ESTRADIOL INDUCES CYTOTOXIC EFFECTS TO HUMAN T-LYMPHOMA (JURKAT) CELLS THROUGH OXIDATIVE STRESS. J. Miss. Acad. Sci. 60 (Suppl. 1), 279–283. 26321773 PMC4550313

[B78] Zhang Q. Wu Y.-Z. Zhang Y.-M. Ji X.-H. Hao Q. (2015). Activation of G-Protein Coupled Estrogen Receptor Inhibits the Proliferation of Cervical Cancer Cells via Sustained Activation of ERK1/2. Cell Biochem Funct 33 (3), 134–142. 10.1002/cbf.3097 25753185

[B79] Zhou L. Yu T. Yang F. Han J. Zuo B. Huang L. (2021). G Protein-Coupled Estrogen Receptor Agonist G-1 Inhibits Mantle Cell Lymphoma Growth in Preclinical Models. Front. Oncol. 11, 668617. 10.3389/fonc.2021.668617 34211844 PMC8239310

[B80] Zweifach A. Lewis R. S. (1993). Mitogen-regulated Ca2+ Current of T Lymphocytes Is Activated by Depletion of Intracellular Ca2+ Stores. Proc. Natl. Acad. Sci. 90 (13), 6295–6299. 10.1073/pnas.90.13.6295 8392195 PMC46915

