# Supplementary material for: The G-Protein–Coupled Estrogen Receptor Agonist G-1 Inhibits Proliferation and Causes Apoptosis in Leukemia Cell Lines of T Lineage
Source: Front Cell Dev Biol. 2022 Feb 14;10:811479. doi: 10.3389/fcell.2022.811479 (PMC8882838; doi:10.3389/fcell.2022.811479)
Supplement: Supplementary file 1 [file Table1.DOCX]

| **Supplementary table S1. Biological effects of G-1** | | | | | | | |
| --- | --- | --- | --- | --- | --- | --- | --- |
| **Biological effect** | **Methodology** | **Model** | **Concentration** | **Time** | **GPER dependent** | | **Reference** |
|  |  |  |  |  | Yes/Not | Method |  |
| **Inhibition of cell viability and proliferation** | Automated Cell Counter | MDA-MB-231 (breast cancer, human) | 1 µM | 72 h | Yes | siGPER | Weißenborn et al., 2014 |
|  |  | IGROV-1 (ovarian epithelial cancer) | 0.5-2 µM | 60 h | N.S. | - | Wang et al., 2013 |
|  |  | SKOV-3 (ovarian adenocarcinoma, human) | 1-2 µM |  |  |  |  |
|  |  | KGN (ovarian granulose cell tumor, human) | 2 µM | 48 h | Not | G-15  4 µM | Wang et al., 2012 |
|  |  | MDA-MB 231 (breast cancer, human) |  | 36 h | Not | G-15  2-8 µM |  |
|  |  | MCF-7 (breast cancer, human) | 1-2 µM | 48 h | N.S. | - | Lv et al., 2017 |
|  |  | SKBr3 (breast adenocarcinoma, human) |  |  |  |  |  |
|  |  | MDA-MB 231 (breast cancer, human) | 0.5-2 µM |  |  |  |  |
|  | [3H]-thymidine incorporation | bEnd.3 (brain microvascular endothelium, mouse) | 3 µM | 48 h | N.S. | - | Holm et al., 2012 |
|  |  | H295R (adenocortical carcinoma, human) | 1 µM | 24-72 h | N.S. | - | Chimento et al., 2015 |
|  | Cell count, trypan blue dye exclusion | K1735-M2 (melanoma, mouse) | 5 µM | 72 h | N.S. | - | Ribeiro et al., 2017 |
|  | WST-8 | Endometriotic stromal cells | 10 µM | 96 h | N.S. | - | Mori et al., 2015 |
|  |  | A549 (lung cancer, human) | 10-100 µM | 48-72 h | N.S. | - | Kurt et al., 2015 |
|  |  | AGS (gastric cancer, human) | 2.5-20 µM | N.S. | Yes | siGPER  G-15  1 µM | Lee et al., 2019 |
|  | Sulforhodamine B (SRB) assay | K1735-M2 (melanoma, mouse) | 1-20 µM | 24-72 h | N.S. | - | Ribeiro et al., 2017 |
|  | CCK-8 kit | SkBr3 (breast adenocarcinoma, human) | 1 µM | 24-48 h | Yes | siGPER | Wei et al., 2014 |
|  |  | MDA-MB 231 (breast cancer, human) |  |  |  |  |  |
|  |  | HCT-116 (colon cancer, human) | 1 µM | 24-72 h | N.S. | - | Liu et al., 2017 |
|  |  | SW480 (colon cancer, human) |  | 48-72 h |  |  |  |
|  |  | Jeko-1 (mantle cell lymphoma, human) | 1-8 µM | 72 h | N.S. | - | Zhou et al., 2021 |
|  |  | Mino (mantle cell lymphoma, human) | 2-8 µM |  |  |  |  |
|  |  | Rec-1 (mantle cell lymphoma, human) |  |  |  |  |  |
|  |  | Granta-519 (mantle cell lymphoma, human) |  |  |  |  |  |
|  | BrdU incorporation | K1735-M2 (melanoma, mouse) | 1-5 µM | 48 h | N.S. | - | Ribeiro et al., 2017 |
|  |  | Caov-3 (ovarian cancer, human) | 1 µM | 24 h | Yes | siGPER/ G-15  1 µM | Han et al., 2021 |
|  |  | Caov-4 (ovarian cancer, human) |  |  |  |  |  |
|  | MTT assay | IGROV-1 (ovarian epithelial cancer, human) | 0.5-2 µM | 48 h | N.S. | - | Wang et al., 2013 |
|  |  | SKOV-3 (ovarian adenocarcinoma, human) | 1-2 µM |  |  |  |  |
|  |  | KGN (ovarian granulose cell tumor, human) | 2 µM | 24 h | Not | G-15  4 µM | Wang et al., 2012 |
|  |  | HEK-293 (embryonic kidney, human) | 1-2 µM | 24-60 h | Not | GPER-negative cell line |  |
|  |  | MCF-7 (breast cancer, human) | 0.1-10 µM | 96 h | Not | G-15  1 µM | Lv et al., 2017 |
|  |  | SKBr3 (breast adenocarcinoma, human) | 1-10 µM |  |  |  |  |
|  |  | MDA-MB 231 (breast cancer, human) | 0.1-10 µM |  |  |  |  |
|  |  | BT-549 (breast cancer, human) | 0.001-10 µM |  |  |  |  |
|  |  | Hs 578T (breast cancer, human) | 0.1-10 µM |  |  |  |  |
|  |  | HCC 1937 (breast cancer, human) | 1-10 µM |  |  |  |  |
|  |  | A549 (lung cancer, human) | 10-100 µM | 48-72 h | N.S. | - | Kurt et al., 2015 |
|  |  |  | 20 µM | 48-72 h | Not | G-15  20 µM |  |
|  |  | MDA-MB 231 (breast cancer, human) | IC_50_: 0.1 µM | 72 h | Yes | GPER siRNA | Weißenborn et al., 2014 |
|  |  | MDA-MB 468 (breast cancer, human) | IC_50_: 0.3 µM |  |  |  |  |
|  |  | H295R (adenocortical carcinoma, human) | 1 µM | 48-72 h | Partially | shGPER | Chimento et al., 2015 |
|  |  | K1735-M2 (melanoma, mouse) | 10 µM | 24 h | Yes | siGPER | Ribeiro et al., 2017 |
|  |  | Caov-3 (ovarian cancer, human) | IC_50_: 2.25 µM | 24 h | N.S. | - | Han et al., 2021 |
|  |  | Caov-4 (ovarian cancer, human) | IC_50_: 2.29 µM |  |  |  |  |
|  | N.S. | 2838c3 (pancreatic ductal adenocarcinoma, murine) | 0.25 µM | 96 h | Yes | G-36  1 µM | Natale et al., 2020 |
|  |  | 6419c5 (pancreatic ductal adenocarcinoma, murine) |  |  |  |  |  |
|  |  | 6499c4 (pancreatic ductal adenocarcinoma, murine) |  |  |  |  |  |
|  |  | PANC-1 (pancreatic ductal adenocarcinoma, human) | 0.5 µM | 4-7  days | N.S. | - |  |
|  |  | HPAC (pancreatic ductal adenocarcinoma, human) |  |  |  |  |  |
|  |  | MIAPaCa-2 (pancreatic ductal adenocarcinoma, human) |  |  |  |  |  |
| **Increase of cell**  **proliferation** | Cell count / hemo-cytometer | BG-1 (ovarian cancer, human) | 0.1 µM | 6 days | Yes | shGPER | Albanito et al., 2007 |
|  |  | 2008 (ovarian cancer, human) |  |  |  |  |  |
|  |  | SKBr3 (breast adenocarcinoma, human) |  |  |  |  |  |
|  | Countess Automated Cell Counter | Cancer associated fibroblasts | 0.1 µM | 5 days | Yes | shGPER  G-15  0.1 µM | Cirillo et al., 2017 |
|  |  | SKBr3 (breast adenocarcinoma, human) |  |  |  |  |  |
|  |  | MDA-MB 231 (breast cancer, human) |  |  |  |  |  |
|  |  | SKBr3 (breast adenocarcinoma, human) | 0.1 µM | 72 h | N.S. | - | Lappano et al., 2019 |
|  | MTT assay | 11z (immortalized epithelial endometriotic cells, human) | 3-10 µM | 72 h | Yes | G-15 30 µM | Imesch et al., 2013 |
|  | CCK-8 kit | Cancer associated fibroblasts isolated from primary breast cancer tissues | 1 µM | 72 h | yes | G-15  1 µM | Luo et al., 2014 |
|  |  | A549 (lung cancer, human) | 0.01-0.1 µM | 48 h | Yes | G-15  0.1-1 µM | Liu et al., 2019 |
|  |  | H1793 (lung cancer, human) |  |  |  |  |  |
|  | Ki-67 | Uterine epithelium derived from ovariectomized female C57Bl6 mice | 0.1-2.4 nmol/mouse | 18 h | Yes | G-15  2.4-27 nmol/  mouse | Dennis et al., 2009 |
|  |  |  | 10 µg/kg |  |  | G-36  50 µg/kg | Dennis et al., 2011 |
| **Cell cycle arrest in G2** | Propidium iodide, flow cytometry | IGROV-1 (ovarian epithelial cancer) | 2 µM | 16 h | N.S. | - | Wang et al., 2013 |
|  |  | SKOV-3 (ovarian adenocarcinoma, human) |  |  |  |  |  |
|  |  | KGN (ovarian granulose cell tumor, human) | 2 µM | 24 h | Not | G-15  4 µM | Wang et al., 2012 |
|  |  | HEK-293 (embryonic kidney, human) | 0.5-2 µM | 16 h | Not | GPER-negative cell line |  |
|  |  | Primary cultured  endometriotic stromal cells | 10 µM | 7 days | N.S. | - | Mori et al., 2015 |
|  |  | MCF-7 (breast cancer, human) | 2 µM | 24 h | N.S. | - | Lv et al., 2017 |
|  |  | SKBr3 (breast adenocarcinoma, human) |  |  |  |  |  |
|  |  | MDA-MB 231 (breast cancer, human) |  |  |  |  |  |
|  |  | H295R (adenocortical carcinoma, human) | 1 µM | 24 h | N.S. | - | Chimento et al., 2015 |
|  |  | SkBr3 (breast adenocarcinoma, human) |  | 48-72 h | N.S. | - | Wei et al., 2014 |
|  |  | HCT-116 (colon cancer, human) |  | 12-72 h | N.S. | - | Liu et al., 2017 |
|  |  | MCF-7 (breast cancer, human) |  | 24-72 h | N.S. | - | Vo et al., 2019 |
|  |  | MDA-MB-231 (breast cancer, human) |  | 24-72 h | N.S. | - | Weißenborn et al., 2014 |
|  |  | MDA-MB-468 (breast cancer, human) |  |  |  |  |  |
|  |  | HeLa (cervical carcinoma, human) | 1 µM | 24 h | N.S. | - | Zhang et al., 2015 |
|  |  | SiHa (cervical carcinoma, human) |  |  |  |  |  |
|  |  | K1735-M2 (melanoma, mouse) | 5 µM | 48 h | N.S. | - | Ribeiro et al., 2017 |
|  |  | Jeko-1 (mantle cell lymphoma, human) | 1 µM | 24 h | N.S. | - | Zhou et al., 2021 |
|  |  | Rec-1 (mantle cell lymphoma, human) |  |  |  |  |  |
|  | BrdU incorporation | Human vascular smooth muscle cells | 1-10 µM | 48 h | N.S. | - | Gui et al., 2015 |
| **Apoptosis** | acridine orange/ethidium bromide | A549 (lung cancer, human) | 20 µM | 72 h | N.S. | - | Kurt et al., 2015 |
|  | TUNEL assay | Human vascular smooth muscle cells | 1 µM | 48 h | Not | G-15  10 µM | Gui et al., 2015 |
|  | Annexin V-FITC/PI, confocal microscopy |  | 1-10 µM | 48 h | Not | G-15  10 µM  siGPER |  |
|  | Annexin V-FITC/PI (7-AAD), Flow cytometry | MCF-7 (breast cancer, human) | 2 µM | 72 h | N.S. | - | Lv et al., 2017 |
|  |  | SkBr3 (breast adenocarcinoma, human) |  |  |  |  |  |
|  |  | MDA-MB 231 (breast cancer, human) |  |  |  |  |  |
|  |  | H295R (adenocortical carcinoma, human) | 1 µM | 24-48 h | N.S. | - | Chimento et al., 2015 |
|  |  | RWPE-1 (prostate epithelial, human) | 2.5 µM | 48 h | N.S. | - | Yang et al., 2017 |
|  |  | Dog prostate epithelial cells |  |  |  |  |  |
|  |  | SkBr3 (breast adenocarcinoma, human) | 1-5 µM | 48 h | N.S. | - | Wei et al., 2014 |
|  |  | MDA-MB 231 (breast cancer, human) |  |  |  |  |  |
|  |  | HCT-116 (colon cancer, human) | 1-5 µM | 48 h | N.S. | - | Liu et al., 2017 |
|  |  | MDA-MB-231 (breast cancer, human) | 1 µM | 24-72 h | N.S. | - | Weißenborn et al., 2014 |
|  |  | MDA-MB-468 (breast cancer, human) |  |  |  |  |  |
|  |  | MCF-7 (breast cancer, human) | 1 µM | 24-72 h | N.S. | - | Vo et al., 2019 |
|  |  | Jeko-1 (mantle cell lymphoma, human) | 1-5 µM | 48 h | Yes | siGPER | Zhou et al., 2021 |
|  |  | Rec-1 (mantle cell lymphoma, human) |  |  | N.S. | - |  |
|  |  | Mino (mantle cell lymphoma, human) |  |  | N.S. | - |  |
| **Microtubule disruption** | α-tubulin immuno-cytochemistry | bEnd.3 (brain microvascular endothelium, mouse) | 0.3-3 µM | 24 h | Not | GPER1 knockout mice | Holm et al., 2012 |
|  |  | HUVEC (umbilical  vein endothelial, human) |  |  |  |  |  |
|  |  | SKOV-3 (ovarian adenocarcinoma, human) | 2 µM | 16 h | N.S. | - | Wang et al., 2013 |
|  |  | Primary cultured  endometriotic stromal cells | 10 µM | 96 h | N.S. | - | Mori et al., 2015 |
|  | β-tubulin immuno-fluorescence | Mino (mantle cell lymphoma, human) |  | 48h | Yes | G-36 | Rudelius et al., 2015 |
|  |  | Granta-519 (mantle cell lymphoma, human) |  |  |  |  |  |
|  | β-tubulin immuno-cytochemistry | MDA-MB 231 (breast cancer, human) | 10 µM | 2 h | N.S. | - | Lv et al., 2017 |
|  |  | MCF-7 (breast cancer, human) |  |  |  |  |  |
|  | α-tubulin and γ-tubulin immuno-cytochemistry | Vascular smooth muscle cells derived from aorta | 1 µM | 24 h | N.S. | - | Gui et al., 2015 |
| **Intracellular calcium mobilization** | Fura-2 / microscopy | MCF-7 (breast cancer, human) | 1 µM | [Ca^2+^]_i_  511 nM | Yes | siGPER | Ariazi et al., 2010 |
|  |  | SKBr3 (breast adenocarcinoma, human) |  | [Ca^2+^]_i_ 1517 | N.S. | - |  |
|  |  | Rat brain microvascular endothelial cells | 10 µM | [Ca^2+^]_i_  286 nM | Yes | G-36  10 µM | Altmann et al., 2015 |
|  |  | Rat preganglionic neurons | 1 µM | [Ca^2+^]_i_  914 nM | Yes | G-36  1 µM | Brailoiu et al., 2013 |
|  | Indo-1 dye | MCF-7  (breast cancer, human) | 0.5-5 µM |  | N.S. | - | Vo et al., 2019 |
|  | Fluo-3/ microscopy | Cancer associated fibroblasts isolated from primary breast cancer tissues | 1 µM | ≈ 1.7 fold rise (transient) | Yes | G-15 1 µM | Luo et al., 2014 |
|  |  | Dog prostate epithelial cells | 2.5 mM | ≈ 1.7 fold rise | N.S. | - | Yang et al., 2017 |
|  | Indo-1 AM, spectro-fluorometer | SKBr3 (breast adenocarcinoma, human) | 50 nM | ≈ 1.7 fold rise | N.S. | - | Nayak et al., 2010 |
|  |  |  | IC_50_ 165 nM | Ca^2+^]_i_  200 nM | Yes | G-36  10 µM | Dennis et al., 2011 |
| **Decrease in ΔΨm** | JC-1, flow cytometry | SKBr3 (breast adenocarcinoma, human) | 1-5 µM | 24 h | N.S. | - | Wei et al., 2014 |
|  |  | HCT-116 (colon cancer, human) |  |  |  |  | Liu et al., 2017 |
|  |  | Jeko-1 (mantle cell lymphoma, human) | 1 µM | 48 h | N.S. | - | Zhou et al., 2021 |
|  |  | Mino (mantle cell lymphoma, human) |  |  |  |  |  |
| **No decrease in ΔΨm** | TMRE, flow  cytometry | MCF-7 (breast cancer, human) | 1 µM | 3-8 h | N.S. | - | Vo et al., 2019 |
| **ROS induction** | DCF, flow cytometry | SKBr3 (breast adenocarcinoma, human) | 1-5 µM | 4 h | N.S. | - | Wei et al., 2014 |
|  |  | HCT-116 (colon cancer, human) |  | 3 h | N.S. | - | Liu et al., 2017 |
|  |  | Mino (mantle cell lymphoma, human) | 1 µM | 24 h | N.S. | - | Zhou et al., 2021 |
|  |  | Jeko-1 (mantle cell lymphoma, human) | 1-5 µM | 24 h |  |  |  |
|  |  | Granta-519 (mantle cell lymphoma, human) |  |  |  |  |  |
| **N.S.: non-specified ΔΨm: mitochondrial membrane potential ROS: reactive oxygen species** | | | | | | | |

| **Supplementary table S2. EC_50_ of biological effects of G-1 in Jurkat cells cultured in different RPMI 1640 media** | | | | | |
| --- | --- | --- | --- | --- | --- |
| **Effect** | | **Time** | **EC_50_ G-1** | | **Statistical significance** |
|  |  |  | **(+) phenol red**  **5% FBS** | **(-) phenol red**  **10% FCS, dialyzed** |  |
| Decrease in cell viability (cell count) | | 48 h | 0.49 ± 0.04 µM | 0.56 ± 0.09 µM | Not |
|  |  | 72 h | 0.38 ± 0.04 µM | 0.45 ± 0.08 µM | Not |
| Cell death (Annexin V/PI assay) | Decrease in cell viability (Q4 gate) | 24 h | 0.62 ± 0.1 μM | 1.09 ± 0.19 μM | Not |
|  | Cell death induction (Q2+Q3 gate) |  | 0.68 ± 0.19 μM | 1.05 ± 0.19 μM | Not |

| **Supplementary table S3. EC_50_ of biological effects of G-1 and G-36 in Jurkat cells** | | | | |
| --- | --- | --- | --- | --- |
| **Effect** | **Time** | **EC_50_ G-1** | | **Statistical significance** |
|  |  | **G-1** | **G-1 + G-36** |  |
| Cell viability (cell count) | 48 h | 0.49 ± 0.04 µM | 0.34 ± 0.11 μM | Yes |
|  | 72 h | 0.38 ± 0.04 µM | 0.25 ± 0.04 μM | Yes |
| Cell proliferation | 72 h | 0.59 ± 0.06 μM | 0.6 ± 0.07 μM | Not |
| Apoptosis induction | 24 h | 0.68 ± 0.19 μM | 0.48 ± 0.11 μM | Not |

| **Supplementary table S4. EC_50_ of biological effects of G-1 in CD4^+^ activated lymphocytes and Jurkat cells** | | | | |
| --- | --- | --- | --- | --- |
| **Effect** | **Time** | **EC_50_ G-1** | | **Statistical significance** |
|  |  | **Jurkat** | **CD4^+^** |  |
| Cell viability (cell count) | 48 h | 0.5 ± 0.04 µM | 0.55 ± 0.17 µM | Not |
|  | 72 h | 0.38 ± 0.04 µM | 0.47 ± 0.05 µM | Yes |
| Cell proliferation | 72 h | 0.59 ± 0.06 μM | 1.28 ± 0.32 µM | Yes |
