# Supplementary figures and images for: The G-Protein–Coupled Estrogen Receptor Agonist G-1 Inhibits Proliferation and Causes Apoptosis in Leukemia Cell Lines of T Lineage
Source: Front Cell Dev Biol. 2022 Feb 14;10:811479. doi: 10.3389/fcell.2022.811479 (PMC8882838; doi:10.3389/fcell.2022.811479)

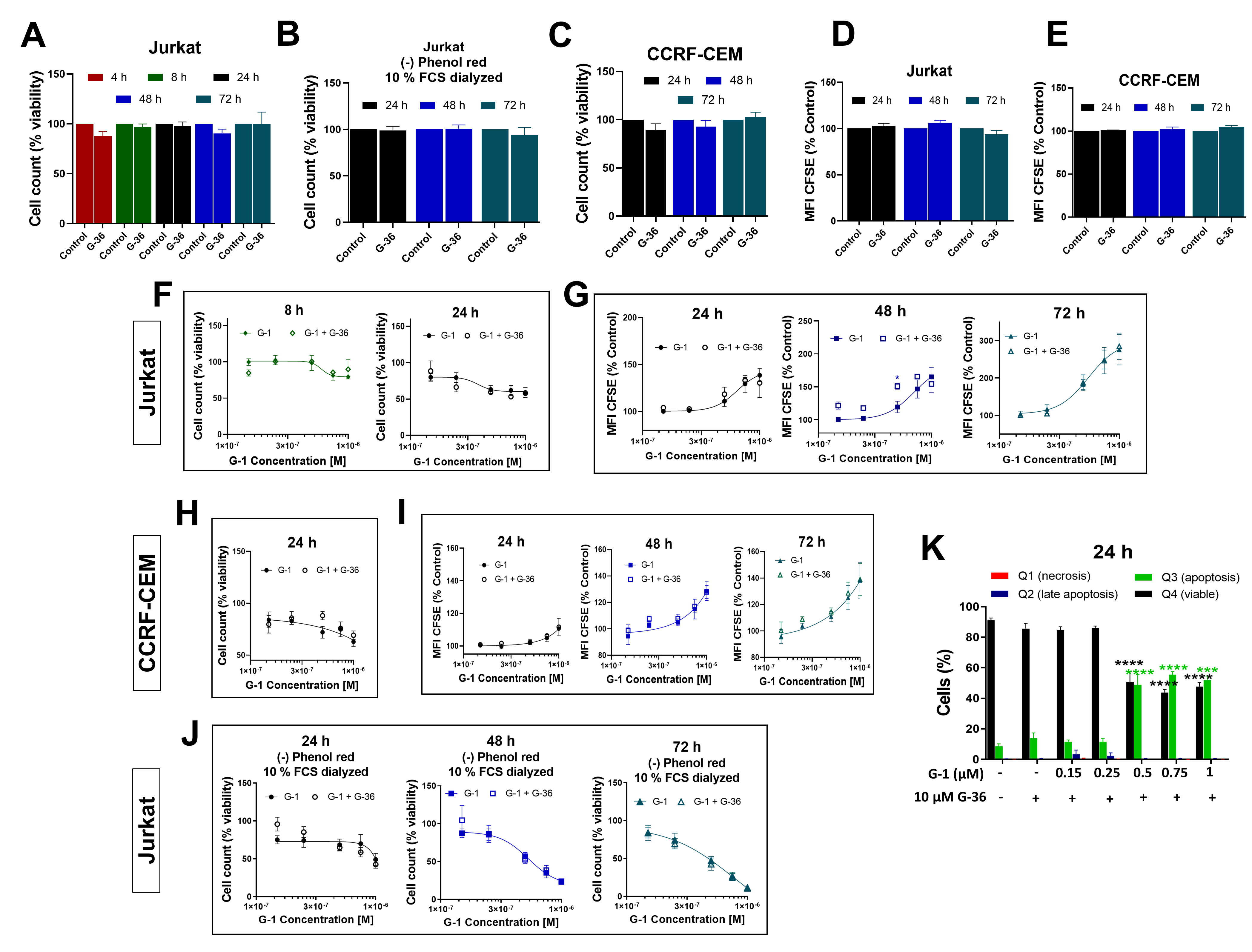

Supplement: Supplementary file 2 [file Image3.TIF]

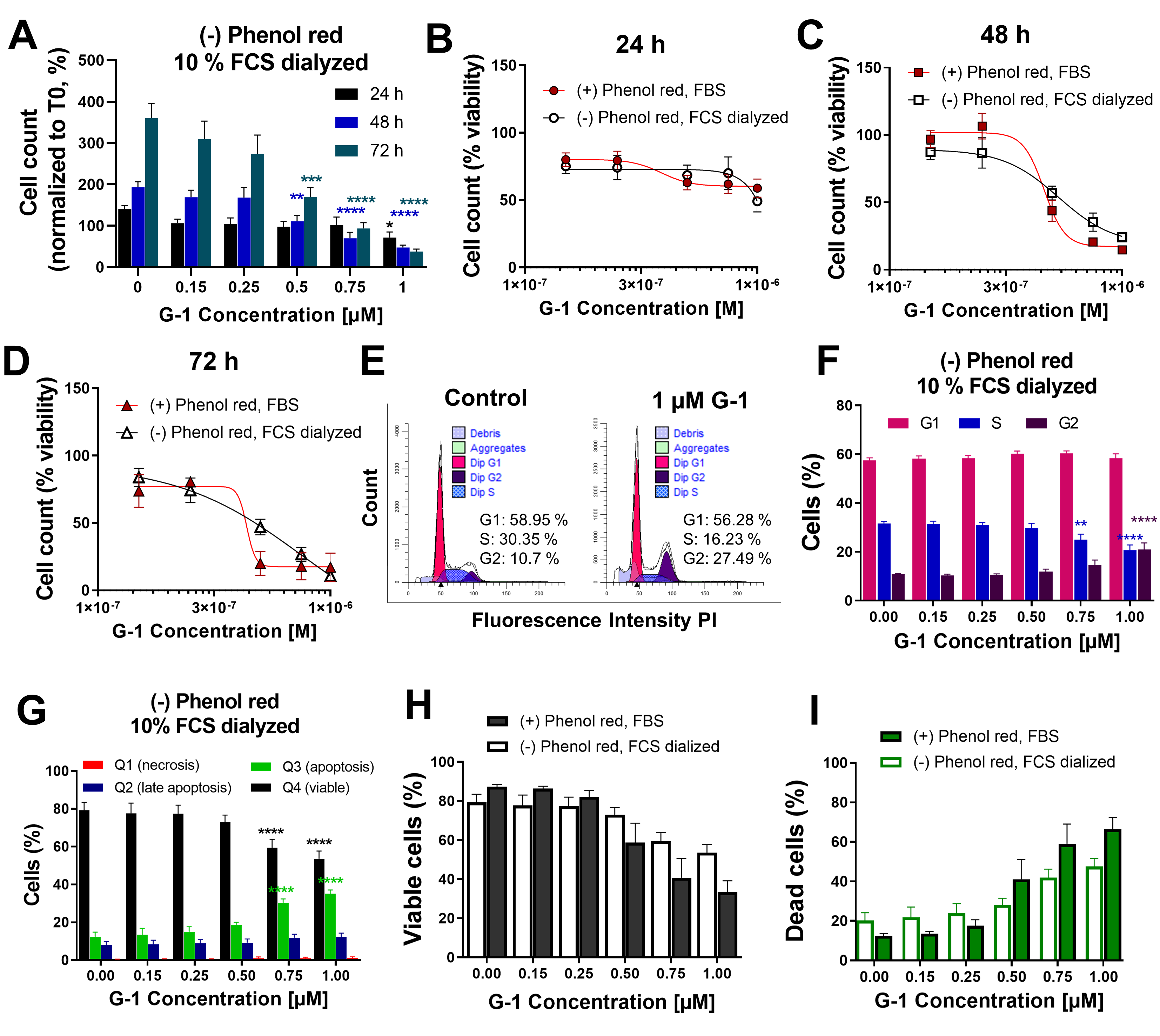

Supplement: Supplementary file 3 [file Image2.TIF]

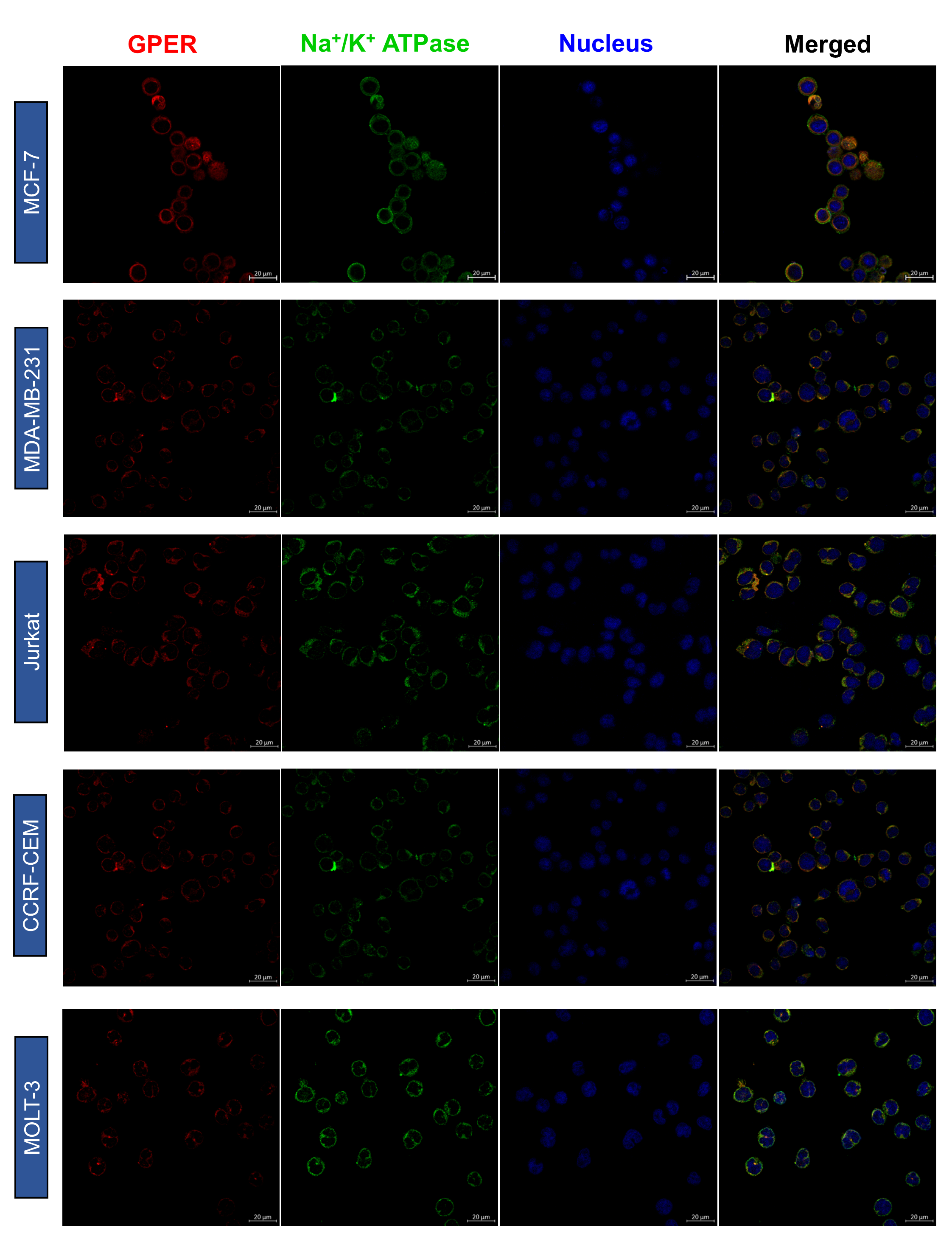

Supplement: Supplementary file 4 [file Image1.TIF]
